# Self‐Assembling Supramolecular Hybrid Hydrogel Beads

**DOI:** 10.1002/anie.201911404

**Published:** 2019-11-27

**Authors:** Carmen C. Piras, Petr Slavik, David K. Smith

**Affiliations:** ^1^ Department of Chemistry University of York Heslington York YO10 5DD UK

**Keywords:** catalysis, gel, self-assembly, supramolecular systems

## Abstract

With the goal of imposing shape and structure on supramolecular gels, we combine a low‐molecular‐weight gelator (LMWG) with the polymer gelator (PG) calcium alginate in a hybrid hydrogel. By imposing thermal and temporal control of the orthogonal gelation methods, the system either forms an extended interpenetrating network or core–shell‐structured gel beads—a rare example of a supramolecular gel formulated inside discrete gel spheres. The self‐assembled LMWG retains its unique properties within the beads, such as remediating Pd^II^ and reducing it in situ to yield catalytically active Pd^0^ nanoparticles. A single PdNP‐loaded gel bead can catalyse the Suzuki–Miyaura reaction, constituting a simple and easy‐to‐use reaction‐dosing form. These uniquely shaped and structured LMWG‐filled gel beads are a versatile platform technology with great potential in a range of applications.

## Graphical abstract

**Bead it**! The combination of a supramolecular gel and alginate can be used to enforce spherical core–shell bead structures onto catalytic gels self‐assembled from low‐molecular‐weight gelators. The system is able to remediate Pd^II^ and reduce it in situ to yield catalytically active Pd^0^ nanoparticles and can also catalyse the Suzuki–Miyaura reaction when loaded with these nanoparticles.

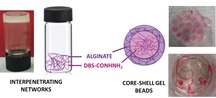

## Introduction

Supramolecular hydrogels self‐assembled from low‐molecular‐weight gelator (LMWG) building blocks in water have seen rapid recent development.^1^ A range of gels has been developed targeting high‐tech applications including regenerative medicine, wound healing, pharmaceutical formulation, optoelectronics, energy storage, and environmental remediation.^2^ The physical features of a gel (for example, stiffness or porosity) as well as the chemical programming inherent within the LMWG scaffold can optimise gels for specific applications. However, in many cases, supramolecular gels suffer from rheological weakness, meaning they simply fill the vessel in which they are formed. This can make it challenging to endow self‐assembled gels with desired shapes and/or structures, yet the ability to shape and pattern such gels would open up new horizons for LMWGs.^3^ Gels with spatially resolved structures could, for example, direct stem‐cell fate in regenerative medicine,^4^ act as vehicles for controlled drug delivery,^5^ or act as patterned conducting gels in integrated soft electronic devices that may ultimately interface with living systems.^6^ A number of strategies have emerged to shape and structure supramolecular gels,^3^ including photopatterning,^7^ 3D printing,^8^ electrochemistry,^9^ and surface‐mediated processes.^10^ Several reports have used controlled diffusion to achieve spatial resolution.^11^ Surprisingly, however, there have been limited reports of a supramolecular gel being formulated as spherical particles. Miravet and co‐workers reported this by dropwise addition of a DMSO solution of the gelator into an anti‐solvent,^12^ while Ulijn and co‐workers used microfluidic methods and a water‐in‐oil emulsion to form gel microspheres.^13^ Spherical nanofibre shells have also been formed via gelator self‐assembly at the interface of oil‐in‐water emulsion microspheres.^14^

**Figure 1:**
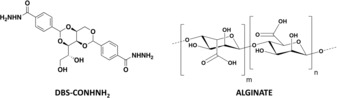
Gelator structures—low‐molecular‐weight gelator (LMWG) DBS‐CONHNH_2_ and polymer gelator (PG) based on alginic acid.

A key strategy that can enhance the behaviour of LMWGs is to form hybrid gels,^15^ combining them with a polymer gelator (PG)—such systems can, for example, combine the responsiveness of a LMWG with the robustness of a PG network, hence enabling more effective shaping and structuring of the LMWG system.^3^ Alginic acid is a fascinating PG—biocompatible, biodegradable, and versatile.^16^ It is a natural polysaccharide composed of β‐d‐mannuronic acid and α‐l‐glucuronic acid units linked through β‐1,4 bonds (Figure 1). Its sodium salt is water‐soluble and forms hydrogels when mixed with multivalent cations (for example, Ca^2+^) by generating ionic interchain bridges. Alginate gel beads can be obtained by dropwise addition of an aqueous alginate solution to CaCl_2_.^17^ This is a well‐known gel system, explored in schools‐level education and exploited in pharmaceutical as well as food sectors.^18^ More complex core–shell alginate beads in which the interior of the bead is filled with a second component can also be made.^19^ However, to the best of our knowledge, there are no examples in which a self‐assembled LMWG has been incorporated within a polymer microgel bead.

**Figure 2:**
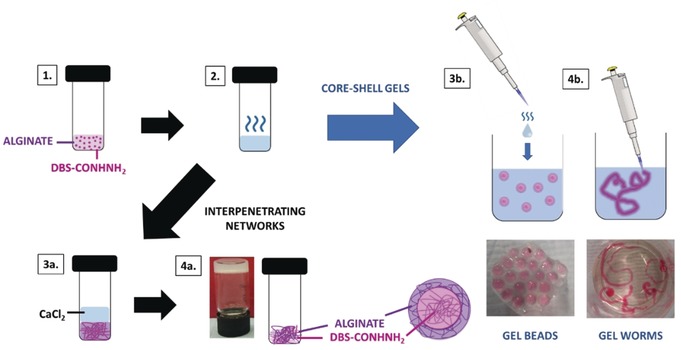
Schematic representation of the preparation of DBS‐CONHNH_2_/alginate multicomponent gels. 1) DBS‐CONHNH_2_ is suspended in an aqueous solution of sodium alginate. 2) The suspension is heated until complete dissolution of the LMWG. To obtain a hybrid gel with extended interpenetrating networks, the hot solution was left to cool to room temperature, allowing the DBS‐CONHNH_2_ network to form. An aqueous solution of CaCl_2_ (5 %, 1 mL) was then added on top of the gel (3 a) to diffuse in, cross‐link the alginate, and form the second gel network (4 a). Alternatively, the hot solution was added to an aqueous solution of CaCl_2_ dropwise, or as a continuous stream to form gel beads or (3 b) worms/strings (4 b), respectively. Note: The gels were coloured pink using food colouring to be better visualised.

We envisaged a multicomponent gel in which the PG network would effectively act as a spherical mould to constrain LMWG self‐assembly. To achieve this spatial control, we decided to combine the alginate PG with 1,3:2,4‐di‐(4‐acylhydrazide)‐benzylidenesorbitol (DBS‐CONHNH_2_, Figure 1), a thermally responsive gel, demonstrated to be biocompatible and with potential applications ranging from environmental remediation and catalysis to drug formulation and tissue engineering.^20^ In this paper, we report how combining the PG and LMWG gives a multicomponent system. The two networks can be spatially organised either a) as an extended standard vial‐filling gel with interpenetrating networks, or b) as well‐defined core–shell‐structured gel beads (or worms). Surprisingly, given the many uses of alginate and the versatility of LMWGs, reports of hybrid gels combining an alginate PG with LMWGs are rare, and in all cases, standard gels were made.^21^ To the best of our knowledge, this is the first time a PG has imposed spherical shape onto an LMWG, thus yielding core–shell supramolecular gel beads (Figure 2).

## Results and Discussion

The LMWG, DBS‐CONHNH_2_, was synthesized as previously reported.20a This LMWG forms hydrogels at low concentrations (0.3 % wt/vol) using a heat–cool cycle. Low‐viscosity sodium alginate PG is commercially available and, as described above, can be triggered by CaCl_2_ to form gels. Since the two gels have orthogonal assembly methods, we reasoned a specific spatial arrangement of the two networks within the hybrid LMWG/PG gel could be imposed (Figure 2).

Initially, to demonstrate the orthogonality of the two gelators, the DBS‐CONHNH_2_/alginate interpenetrating network hydrogel was obtained using a stepwise approach (Figure 2, panel 4 a) combining an aqueous suspension of DBS‐CONHNH_2_ (0.3 % wt/vol) with sodium alginate (0.5 % wt/vol). Heating ensured complete dissolution of the LMWG, with gelation being triggered on cooling. Once the DBS‐CONHNH_2_ gel had formed, an aqueous solution of CaCl_2_ (5 % wt/vol, 1 mL) was added to the top of the gel and allowed to diffuse through it, causing cross‐linking and gelation of the alginate. The gel properties are briefly summarised below.

Macroscopically, the thermal stability, evaluated via a simple tube‐inversion method, indicated that alginate increased the gel–sol transition temperature (*T*_gel_) from 86 °C for DBS‐CONHNH_2_ gel (0.4 % wt/vol) to >100 °C, that is, the alginate PG network improves thermal stability. In terms of mechanical properties, oscillatory rheology using parallel plate geometry indicated that the DBS‐CONHNH_2_ hydrogel (0.4 % wt/vol) has an elastic modulus (*G′*) of 800 Pa, the alginate gel (0.4 % wt/vol) has a *G′* of 490 Pa, whereas two‐component gels at similar loadings displayed higher *G′* values (>5000 Pa)—the two networks thus support each other, increasing stiffness (Supporting Information, Table S2, Figures S15–S23). Similar effects are frequently observed for interpenetrated network polymer gels.^22^ Furthermore, hybrid gels obtained using low alginate concentrations were more resistant to strain than gels formed by the individual components, with a linear viscoelastic region (LVR) that extends up to ca. 50 % strain in contrast to ca. 6.5 % for an equivalent amount of calcium alginate and 25 % for the gel formed only by DBS‐CONHNH_2_. Higher alginate concentrations made the hybrid gels more brittle. The loading of alginate thus allows optimisation between *G′* (stiffness) and resistance to strain (brittleness). Tunable mechanical properties of this type are of great use in cell‐culture applications.^4^

On the nanoscale, scanning electron microscopy (SEM) and transmission electron microscopy (TEM) indicated that DBS‐CONHNH_2_ and calcium alginate both formed extended nanofibres, ca. 20–40 nm and 40–60 nm in diameter, respectively (Figures S8 and S9). The two‐component interpenetrated network gel exhibited long nanofibers with diameters of ca. 20–50 nm, consistent with both LMWG and PG networks being present—the networks could not be differentiated because of their relatively similar fibre diameters.

On the molecular scale, IR spectroscopy (Figure S2) indicated that the alginate O−H stretching frequency (3390 cm^−1^) shifted to 3370, 3362, and 3351 cm^−1^ in the two‐component gel obtained using 1.0 %, 0.5 %, and 0.3 % wt/vol of alginate, respectively, combined with 0.3 % wt/vol of DBS‐CONHNH_2_. As the relative loading of LMWG increases, the alginate O−H band thus progressively shifts to smaller wavenumbers, suggesting non‐covalent interactions between DBS‐CONHNH_2_ and alginate in the hybrid gel, which provides evidence for interaction between the networks, in support of rheology. In the presence of alginate, the O−H (3282 cm^−1^) and N−H (3167 cm^−1^) stretching bands of DBS‐CONHNH_2_ were broadened, also supportive of interactions with the alginate network.

**Figure 3:**
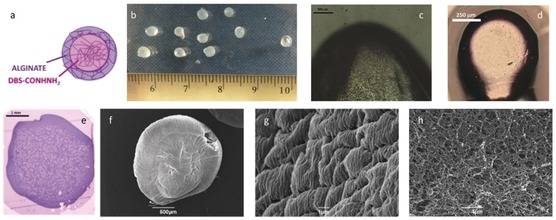
Images of hybrid DBS‐CONHNH_2_/alginate gel beads. a) Schematic diagram of a bead; b) Photograph of beads (droplet size 20 μL) adjacent to a ruler (scale in cm); c)–e) Optical microscopy of the cross‐section of a bead. c) Droplet size 20 μL, scale bar 500 μm, d) droplet size 1 μL, scale bar 150 μm, e) Bead embedded in resin and coloured using toluidine blue (scale bar 1 mm); f) SEM image of a bead (scale bar 500 μm); g) SEM image of the surface of a bead (scale bar 1 μm); h) SEM image of the interior cross‐section of a bead (scale bar 1 μm).

Having determined that these two gel networks could indeed be assembled in a stepwise manner, we then targeted LMWG/PG hybrid gels with spatially constrained organisation—specifically core–shell gel beads (Figure 2, panel 3 b, and Figure 3 a). We used the same quantities of DBS‐CONHNH_2_ and sodium alginate, but after heating, the resulting hot solution was added dropwise to aqueous CaCl_2_ (5 % wt/vol). Small gel beads (Figure 3 b) rapidly formed on calcium‐induced cross‐linking of the alginate chains, with simultaneous self‐assembly of DBS‐CONHNH_2_ on cooling. The beads were then quickly removed from the mixture. To make the method reproducible and obtain gel beads of similar dimensions, each gel bead was prepared by adding 20 μL of hot alginate/DBS‐CONHNH_2_ solution. We combined DBS‐CONHNH_2_ (0.3 % wt/vol) with different alginate concentrations and established that 0.5 % wt/vol was the minimum PG concentration required to isolate spheroidal gel beads. They could also be made with higher alginate concentrations (that is, 0.75 and 1.0 % wt/vol), but at lower concentrations (≤0.3 % wt/vol), they were more irregular.

The gel beads had diameters of 3.0–3.6 mm (Figure 3 b). The theoretical diameter (*d*) of a sphere of a known volume (*V*) is obtained using: $d=2\sqrt[{3}]{3V/4\pi }$ . Since for each gel bead, a volume of 0.020 mL (=0.02 cm^3^) was used, spheres of 0.336 cm (3.36 mm) diameter were predicted—the observed bead diameters therefore agree with expectations. The diameter was varied by simply changing the volume of liquid added dropwise to CaCl_2_. Using 5 and 1 μL volumes, we obtained gel beads with diameters of 1.6–2.0 mm and 0.75–1.0 mm, respectively (Figure 3 d). Simple optical microscopy clearly indicated core–shell structures (Figures 3 c and d). Further optical microscopy on the gel beads cut in half, embedded in resin and dyed with toluidine blue, indicated a clear difference between the interior volume and the outer shell in the hybrid gel‐bead cross‐section (Figure 3 e). This was not seen for the beads formed from alginate alone (Figure S7).

SEM (Figure 3 f) provided insight into the nanoscale morphologies of the bead surface (Figure 3 g) and cross‐section (Figure 3 h). Samples were prepared by freeze‐drying to minimise drying effects. DBS‐CONHNH_2_/alginate and alginate gel‐bead surfaces appeared wrinkled (Figures 3 g, S12, and S13) and densely packed, consistent with a highly cross‐linked calcium alginate shell. The SEM image of the cross‐section of the hybrid gel beads shows an extended nanofibrillar network in the core of the bead (Figure 3 h), consistent with incorporation of the LMWG in self‐assembled form inside the bead.

By varying the mode of addition of the hot solution containing the LMWG/PG mixture to CaCl_2_, the gels could be formed into other shapes, for example, worms (Figure 2, panel 4 b). Worms were prepared by adding the hot alginate/DBS‐CONHNH_2_ solution to the CaCl_2_ solution as a thin stream rather than dropwise. Stupp and co‐workers also used calcium chloride solution to induce the formation of gel worms,^23^ although in their case, a peptide amphiphile was used as the self‐assembling unit which itself was stiffened as a result of interactions with calcium ions—no PG was present to mediate the process.

Our data are consistent with a model in which, as the hot solution is added dropwise into calcium chloride, the periphery is rapidly converted into a calcium alginate cross‐linked shell. As the system cools, the LMWG then assembles within this sphere (or worm) to form a self‐assembled gel interior. In this way, the calcium alginate PG effectively acts as a vessel or mould that contains the self‐assembling LMWG. This prevents the LMWG from uncontrolled assembly into an extended vial‐filling gel, and hence yields discrete shaped and structured LMWG/PG objects.

To quantify the amount of DBS‐CONHNH_2_ incorporated into each gel bead, a simple ^1^H NMR experiment was used. Ten beads were isolated and dried under vacuum. The resulting solid was dissolved in [D_6_]DMSO, dissolving all the DBS‐CONHNH_2_ but not alginate, so the LMWG can be determined by ^1^H NMR. Acetonitrile (CH_3_CN) was used as an internal standard, and the concentration of LMWG was hence calculated by comparing the integrals of relevant resonances (DBS‐CONHNH_2_ aromatic protons: δ=7.53 and 7.83 ppm, and the acetonitrile CH_3_ group: δ=2.09 ppm; Figure S1). In principle, 50 gel beads (20 μL volume each) could be prepared from 1 mL of water containing DBS‐CONHNH_2_ (0.3 % wt/vol, 6.32 μmole) and sodium alginate (0.5 % wt/vol). If the DBS‐CONHNH_2_ was fully incorporated and evenly distributed into the gel beads, each bead should contain ca. 0.12 μmole of LMWG. The NMR study showed that the amount of LMWG in each gel bead was ca. 0.11 μmole. This experiment was highly reproducible and we therefore reason that >90 % of the LMWG is incorporated within the LMWG/PG gel beads, demonstrating the efficiency of this fabrication method.

IR spectroscopy (Figures S2 and S3) indicated that in the hybrid gel beads, the O−H and N−H stretching bands of DBS‐CONHNH_2_ were broadened, and the alginate O−H stretching frequency shifts from 3390 cm^−1^ to 3352 and 3341 cm^−1^ (with 1.0 and 0.5 % wt/vol of alginate, respectively). These shifts are similar to those described for the interpenetrated gels described above.

To demonstrate one possible use of these spatially constrained hybrid gels, we explored catalysis. Gels have great potential in catalysis as a result of their solvated nature and the ability of small‐molecule reagents to diffuse through them—indeed, gels can be considered intermediate between homogeneous and heterogeneous catalytic systems, combining the advantages of both.^24^ We recently reported that DBS‐CONHNH_2_ hydrogels achieve efficient in‐situ reduction of precious metals to yield gels with embedded metal nanoparticles (NPs).20c Furthermore, gels with embedded palladium nanoparticles (PdNPs) can catalyse Suzuki–Miyaura cross‐coupling reactions.^25^ We were interested to see if the presence of alginate affected Pd uptake or catalysis and demonstrate advantages of the gel bead formulation.

**Figure 4:**
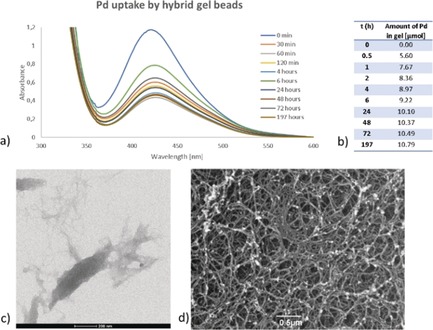
a) UV/Vis spectra of Pd uptake by Pd hybrid gel beads (3.00 mg DBS‐CONHNH_2_ in 0.5 mL H_2_O, 0.5 mL 1 % alginate, 1 mL 5 % CaCl_2_); b) Amount of Pd embedded within the gel beads; c) TEM image of hybrid gel beads with PdNPs, scale bar 200 nm; d) SEM image of hybrid gel beads with PdNPs, scale bar 0.5 μm.

Gel samples were left to interact with aqueous PdCl_2_ (3 mL, ca. 5 mm), and at specified times, the supernatant PdCl_2_ was analysed by UV/Vis spectroscopy (Figures 4 a and S25–S27) to determine the remaining concentration of Pd^II^ and thus the amount of Pd incorporated within the gel. The calcium alginate gel itself could also incorporate Pd^II^, in agreement with literature reports.^26^ However, based on the colour of the Pd‐loaded calcium alginate gel being yellow, the incorporated Pd is clearly still in the oxidation state +2 (Figure S30). On the contrary, when DBS‐CONHNH_2_ was present in the hybrid gel, the gel changed to dark brown/black suggesting not only Pd^II^ incorporation but in‐situ reduction to Pd^0^ (Figure S30). When the gel was prepared as hybrid gel beads, Pd uptake was faster compared with the extended interpenetrating network gel in vials, presumably due to the greater relative surface area of the beads—a black colour, consistent with reduction of Pd^II^ to Pd^0^ NPs, was again observed. The total amount of Pd within the DBS‐CONHNH_2_/alginate gels was ca. 33 % of that in our previously prepared DBS‐CONHNH_2_/agarose gels.^25^ This agrees with the proposal above that there are interactions between DBS‐CONHNH_2_ and alginate networks—we suggest these somewhat limit the ability of the acylhydrazide to reduce Pd^II^ to Pd^0^. The formation of PdNPs was confirmed by TEM and SEM (Figures 4 c,d, S28, and S29), with the NPs being attached to the gel fibres (where the acyl hydrazide groups are located) and mostly spherical with diameters <5 nm.

**Scheme 1:**
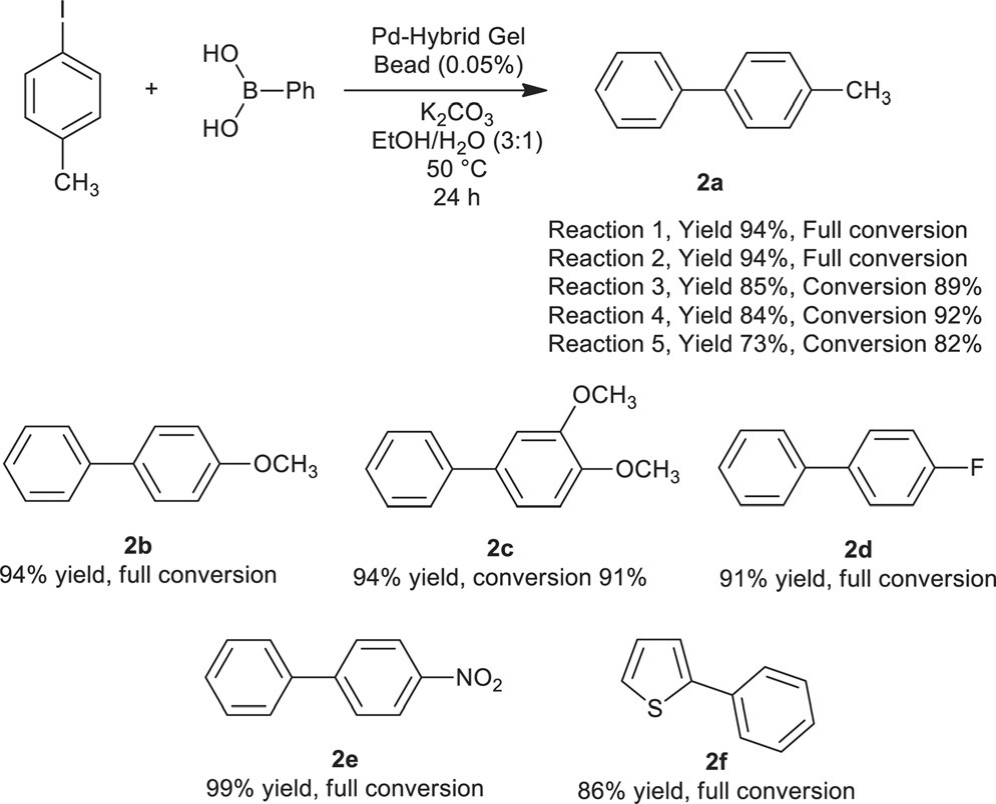
Suzuki cross‐coupling reaction using a single Pd‐hybrid gel bead. Reaction conditions: 4‐iodoarene (0.80 mmol), phenylboronic acid (0.96 mmol), K_2_CO_3_ (1.60 mmol), EtOH (3 mL), H_2_O (1 mL), and Pd hybrid gel bead (prepared using 1 mL of 1 % alginate; containing ≈0.05 mol % of Pd). The reaction was carried out without stirring at 50 °C for 24 h. Compound **2 a** was made using a single gel bead recycled five times. Conversions determined by ^1^H NMR.

The catalytic activity of DBS‐CONHNH_2_/alginate gels loaded with PdNPs was studied in a standard Suzuki–Miyaura cross‐coupling reaction between 4‐iodotoluene and phenylboronic acid (0.8 mmol scale).^25^ When we used the whole amount of extended interpenetrating network DBS‐CONHNH_2_/alginate gel (3 mg of DBS‐CONHNH_2_ and 1 mL of 1 % alginate) containing ca. 12 μmol of Pd (1.2 mol %) and performed the reaction in the vial in which the gel was formed, we obtained the desired product **2 a** in 98 % yield after stirring at 50 °C for 24 h—however, we note that under these stirring conditions, the gel was broken down. The reaction was then performed without stirring, using just one single hybrid gel bead as a catalyst (ca. 0.4 μmol of Pd, 0.05 mol %). Pleasingly, product **2 a** was obtained in 99 % yield after 24 h. This clearly demonstrates that these hybrid gel beads have high catalytic potential, and that even a single bead can effectively catalyse the Suzuki–Miyaura reaction (molar ratio of catalyst:reagent=1:2000). The conversions obtained using these DBS/CONHNH_2_/alginate hybrid LMWG/PG gel beads (99 % after 24 h using 0.05 mol % Pd) were very similar to those obtained and reported previously using simple DBS‐CONHNH_2_/agarose hybrid gels (93 % after 18 h with 0.05 mol % Pd), suggesting encapsulation within the gel bead is not having an adverse effect on catalytic performance. To demonstrate some scope of these catalytic gel beads, we also performed reactions with a range of other substrates to give **2 b**–**2 f** in good yield (Scheme 1).

Although the PdNP gels showed excellent catalytic activity, they could not be easily removed from the reaction. We attempted to increase their mechanical stability by increasing the amount of alginate (1 mL of 2 % alginate solution) or using high‐viscosity alginate (1 mL of 1 % alginate solution), but the gel bead could not be removed with a spatula after reaction. We therefore simply left the gel bead in the reaction vial, removed the reaction mixture by a Pasteur pipette, washed the gel bead with diethyl ether and water, and charged the reaction vial with fresh starting materials. In this way, it was possible to re‐use a single Pd‐hybrid gel bead five times (Scheme 1), with high conversion and yield, pleasingly demonstrating the recyclabillity and re‐use of these gel beads.

We tested the Pd‐leaching from these beads (Supporting Information, Section S11) and found some palladium was leached into the solvent during reaction. At the end of the reaction, if the solution was removed from the bead and charged with different Suzuki‐reaction substrates, then the second Suzuki reaction would proceed to >90 %, even in the absence of the bead. Leaching is more pronounced than from our previously reported agarose hybrid hydrogels (34 % Suzuki coupling achieved using the filtrate).^25^ We suggest that this results from Pd in the alginate shell being less effectively bound and the larger relative surface area of the small beads used here. Nonetheless, a significant amount of Pd is clearly retained within the beads because, as described above, they can be re‐used in the reaction five times (an overall molar ratio of catalyst:reagent=1:10 000) and the gel beads can definitely be considered as the source of Pd required for the reaction.

Overall, it is evident the LMWG retains its fundamental Pd remediation and catalysis properties within these shaped and structured core–shell beads. As such, this fabrication method is a very simple way of imposing shape onto an active and functional self‐assembled gelator. The core–shell hybrid gel beads are a straightforward and efficient dosing form for these catalytic gels—one single bead can be placed into the reaction vessel to facilitate the Suzuki–Miyaura cross coupling and the product is easily extracted. This would therefore be an effective way of supplying catalytic gels in a simple physical form, which is easy for the end‐user to employ in synthesis. This clearly demonstrates the advantage of combining a functional LMWG with a PG that plays the role of imposing defined shape and structure onto the overall system.

## Conclusion

In conclusion, we report spatial control over a LMWG assembly by combining a LMWG (DBS‐CONHNH_2_) and a PG (alginate) to form spherical core–shell gel‐bead structures (or worms). Combining alginate with DBS‐CONHNH_2_ enhances thermal stability and rheological performance. The mechanical properties of the hybrid gel can be tuned by the alginate concentration, giving gels with a range of stiffnesses and optimisable strain resistance, suggesting potential applications in, for example, cell culture. The LMWG (DBS‐CONHNH_2_) retains its unique properties within the hybrid gel, such as the ability to reduce Pd^II^ to Pd^0^ NPs in situ, thus creating catalytic gel beads. Gel beads could be simply added into a Suzuki–Miyaura reaction, with just a single bead being able to facilitate the reaction. The ease of dosing these shaped and structured gel beads demonstrates an advantage of this approach—fusing the function of an active LMWG with the shaping potential of a PG. We suggest that this hybrid‐gel approach using calcium alginate to form gel beads should be broadly applicable to a wide range of different LMWGs in order to formulate them into gel spheres, and work in this direction is currently underway in our laboratories. This is therefore a very simple, highly versatile platform technology that could see widespread use in extending the range of LMWG applications. In addition to working on extending the range of gelators incorporated within these beads, we are also investigating further control to yield micro/nano‐sized gel beads, and demonstrating the full potential scope of encapsulated shaped and structured LMWG materials, thus opening up even more wide‐ranging new applications.

## Conflict of interest

The authors declare no conflict of interest.

## Supporting information

As a service to our authors and readers, this journal provides supporting information supplied by the authors. Such materials are peer reviewed and may be re‐organized for online delivery, but are not copy‐edited or typeset. Technical support issues arising from supporting information (other than missing files) should be addressed to the authors.

Supplementary
