# Supplementary material for: Self‐Assembling Supramolecular Hybrid Hydrogel Beads
Source: Angew Chem Int Ed Engl. 2019 Nov 27;59(2):853–9. doi: 10.1002/anie.201911404 (PMC6973155; doi:10.1002/anie.201911404)
Supplement: Supplementary file 1 — Supplementary [file ANIE-59-853-s001.pdf]

Angewandte Chemie
Eine Zeitschrift der Gesellschaft Deutscher Chemiker

Supporting Information

Self-Assembling Supramolecular Hybrid Hydrogel Beads

*Carmen C. Piras, Petr Slavik, and David K. Smith**

anie_201911404_sm_miscellaneous_information.pdf

SUPPORTING INFORMATION

S1 General experimental methods

S2 Gel preparation

S3 NMR assays

S4 Infrared (IR) spectroscopy

S5 Optical microscopy

S6 Transmission and Scanning Electron Microscopy (TEM and SEM)

S7 Thermal stability studies

S8 Rheology

S9 Uptake of Pd(II)

S10 Suzuki-Miyaura cross-coupling reaction

S11 Pd Leaching Experiment

S12 References

S1 General Experimental Methods

All compounds used in synthesis and analysis were purchased from standard commercial suppliers and used as received. The alginate employed in all the experiments was bought from Sigma Aldrich as sodium salt (2% viscosity). The synthesis of DBS-CONHNH$_2$ was performed in good yields applying previously reported methods.[1,2] ^{1}H NMR spectra were recorded using a Jeol 400 spectrometer (^{1}H 400 MHz). Samples were prepared in DMSO-d$_6$ and chemical shifts (δ) are reported in parts per million (ppm). IR spectra of xerogels were recorded on a PerkinElmer Spectrum Two FT-IR spectrometer. Optical microscopy images were obtained using a Zeiss Axiocam camera on a Zeiss stereo microscope. SEM images were taken using a JEOL JSM-7600F field emission SEM. TEM images were obtained on a FEI Tecnai 12 G^2 fitted with a CCD camera. Fibre sizes and gel bead diameters were measured using the *ImageJ* software. T_{gel} values were obtained using a high precision thermoregulated oil bath using the tube inversion method and were recorded in triplicate. Rheology was measured on a Malvern Instruments Kinexus Pro+ Rheometer fitted with a 20 mm parallel plate geometry. UV-vis spectra were collected on a UV-2401 PC spectrophotometer.

S2. Gel preparation

S2.1 DBS-CONHNH$_2$ gels. DBS-CONHNH$_2$ (0.3 or 0.4% wt/vol) was suspended in water (1 mL). The suspension was sonicated to help the dispersion of the solid particles and then heated until complete dissolution of the compound. The sample was left undisturbed to cool, allowing gel formation in few minutes.

S2.2 Alginate gels. Alginate gels were prepared by adding a $CaCl_2$ solution (5.0% wt/vol – 1 mL) to an aqueous alginate solution (0.4-1.3% wt/vol). Gelation occurred immediately. The excess of $CaCl_2$ solution was then removed and the gels were washed with water multiple times.

S2.3 DBS-CONHNH$_2$/alginate two-component gels (extended interpenetrating networks). DBS-CONHNH$_2$ (0.3% wt/vol in 1 mL final total volume) was suspended in water (0.5 mL) and sonicated to help the dispersion of the solid particles. An aqueous alginate solution (1.0% wt/vol - 0.5 mL) was then added. The amount of alginate and water was adjusted depending on the desired final concentration of the polymer in the different experiments. The resulting suspension was heated

until complete dissolution of the DBS-CONHNH$_2$. The sample was left undisturbed for few hours to allow the formation of the DBS-CONHNH$_2$ network. A solution of $CaCl_2$ (5.0 % wt/vol – 1 mL) was then added on top of each gel to crosslink the alginate chains for 30 min. The excess of $CaCl_2$ solution was then removed and the gels were washed with water multiple times.

S2.4 Alginate gel beads. Alginate gel beads were prepared by dropwise addition (20 μL/drop) of an aqueous alginate solution (0.8% wt/vol) to a $CaCl_2$ solution (5.0% wt/vol). The obtained beads were collected by filtration and washed with water multiple times.

S2.5 DBS-CONHNH$_2$/alginate two-component gel beads. DBS-CONHNH$_2$ (0.3% wt/vol in 1 mL final total volume) was suspended in water (0.5 mL) and sonicated to help the dispersion of the solid particles. An aqueous alginate solution (1.0% wt/vol - 0.5 mL) was subsequently added. The resulting suspension was heated until complete dissolution of the DBS-CONHNH$_2$. The hot solution was then added dropwise (20 μL/drop) to a $CaCl_2$ solution (5.0% wt/vol). The obtained beads were collected by filtration and washed with water multiple times.

S3 NMR assays

^{1}H NMR was employed to quantify the exact amount of DBS-CONHNH$_2$ incorporated into each DBS-CONHNH$_2$/alginate gel bead. The gel beads used for this experiment were prepared by combining DBS-CONHNH$_2$ (0.3 % wt/vol) and alginate (0.5 % wt/vol) in water (1 mL) as described in Section S2.5. Ten gel beads were isolated and dried under high vacuum. The resulting solid was dissolved in DMSO-d$_6$ (0.7 mL), and acetonitrile (2.0 μL) was added as an internal standard. To make sure that all the DBS-CONHNH$_2$ was dissolved, the sample was ground and then sonicated for 30 min. The ^{1}H NMR spectrum was recorded and the concentration of the LMWG calculated by comparison of the integrals of relevant peaks (DBS-CONHNH$_2$ aromatic peaks δ = 7.53 and 7.83 ppm) to that of acetonitrile (δ = 2.09 ppm). To ensure the results were reproducible, this experiment was performed on three different batches of gel beads. It is noted that due to the low solubility of alginate in DMSO-d$_6$, the alginate peaks were not visible.

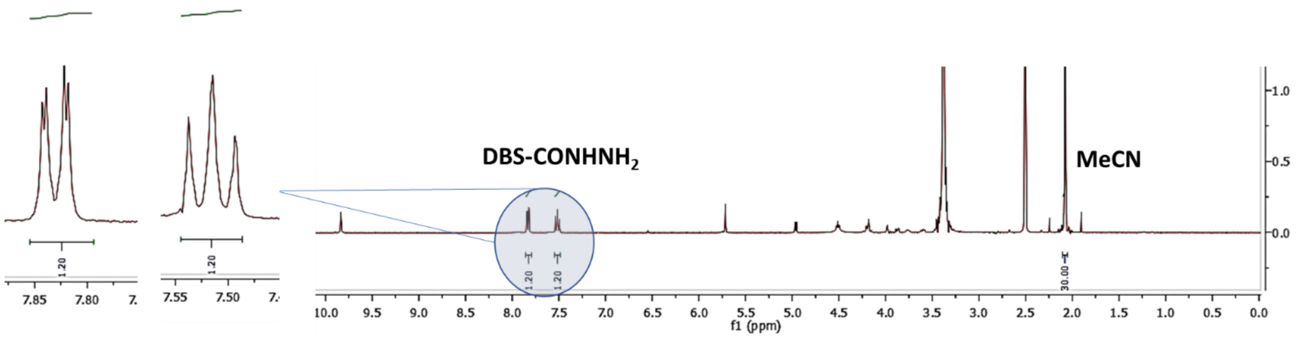

Figure S1. ^{1}H NMR of DBS-CONHNH$_2$ incorporated into ten DBS-CONHNH$_2$/alginate multicomponent gel beads.

S4 Infrared (IR) spectroscopy

Xerogel samples for infrared were prepared by removing the solvent from the gels under high vacuum. A small amount of the resulting powder was placed into the infrared spectrophotometer and the spectra recorded in the range of 450-4000 cm^{-1}.

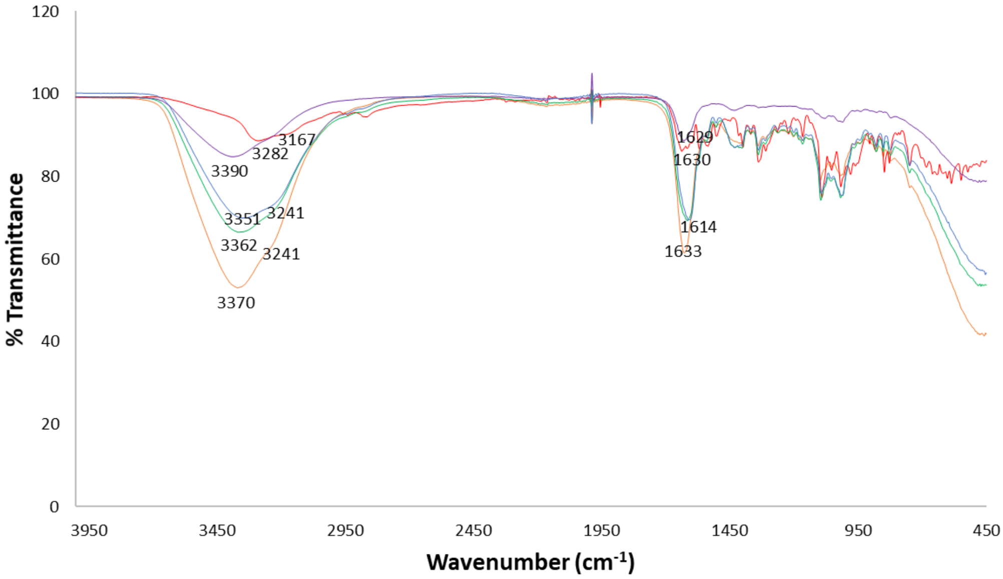

Figure S2. IR spectra of xerogels obtained from DBS-CONHNH$_2$ gel (0.4% wt/vol, red line), alginate gel (0.8% wt/vol, purple line) and DBS-CONHNH$_2$/alginate two-component extended interpenetrating gel containing 0.3% wt/vol of DBS-CONHNH$_2$ and 0.3% wt/vol alginate (blue line), 0.5% wt/vol alginate (green line) and 1.0% wt/vol alginate (orange line).

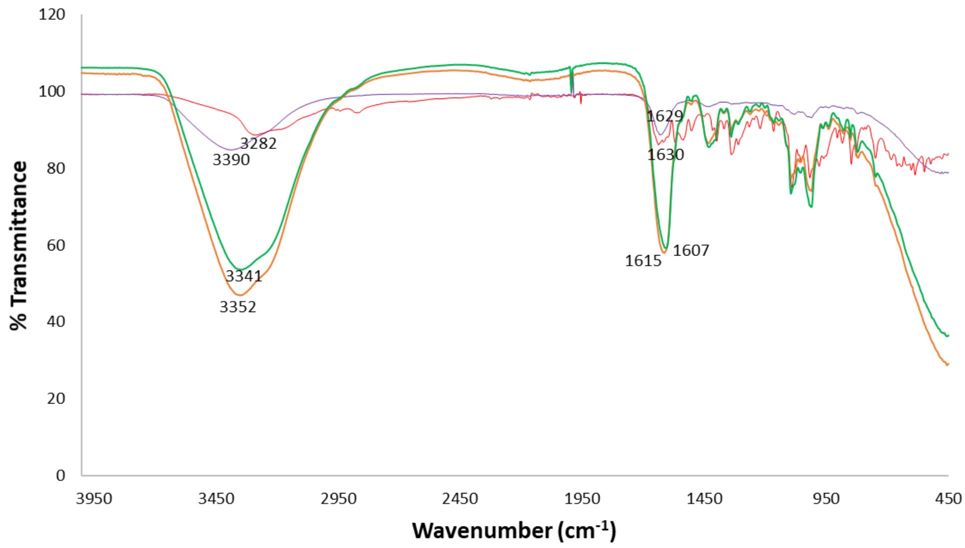

Figure S3. IR spectra of xerogels obtained from DBS-CONHNH$_2$ gel (0.4 % wt/vol, red line), alginate gel (0.8 % wt/vol, purple line) and DBS-CONHNH$_2$/alginate two-component gel beads containing 0.3% wt/vol of DBS-CONHNH$_2$ and 0.5% wt/vol alginate (green line) or 1.0% wt/vol alginate (orange line).

S5 Optical microscopy

Optical microscopy images were collected on a Zeiss stereo microscope. The gel beads were either analysed as such or pre-embedded into resin. These last samples were dehydrated through an ethanol series, then embedded in LR white resin. Sections were 1 μm thick. Once the section was dried on the slide, it was stained with Toluidine Blue (0.6% with 0.3% Na_2CO_3). All the gel beads were prepared using 20 μL volumes (unless otherwise specified) using 0.3% wt/vol of DBS-CONHNH$_2$ and 0.5% wt/vol of alginate (Figure S4).

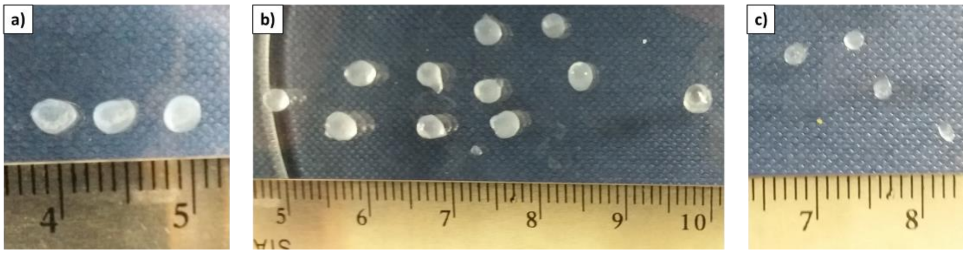

Figure S4. DBS-CONHNH$_2$/alginate two-component gel beads prepared by addition of 20 μL (a and b) and 5 μL (c) volumes into a $CaCl_2$ solution (5%). A ruler was included to show the diameters.

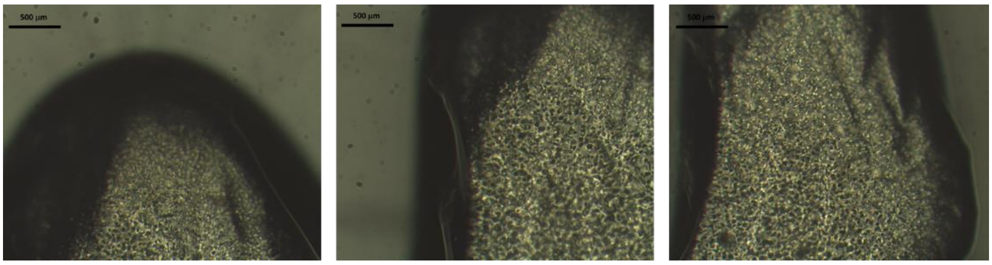

Figure S5. Cross-section of DBS-CONHNH$_2$/alginate two-component gel bead showing core shell morphology. Scale bar: 500 µm.

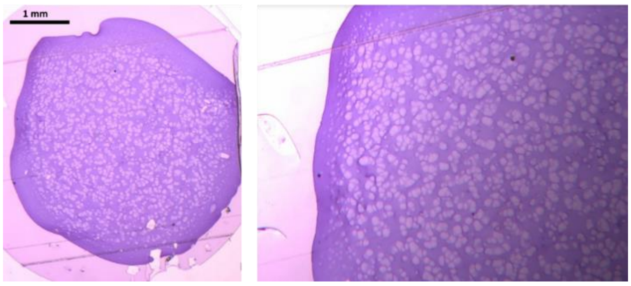

Figure S6. Cross-section of DBS-CONHNH$_2$/alginate two-component gel bead embedded in resin, coloured with toluidine blue. Note core shell morphology and fibrillar interior. Scale bar: 1 mm.

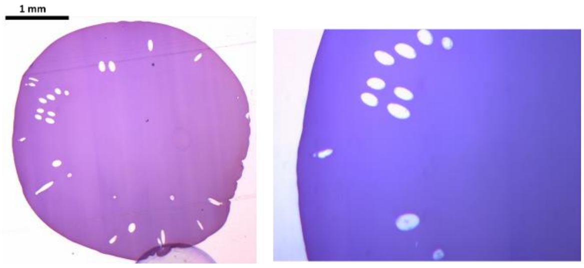

Figure S7. Cross-section of alginate gel bead embedded in resin and coloured using toluidine blue. Scale bar: 1 mm.

S6 Transmission and Scanning Electron Microscopy (TEM and SEM)

S6.1 Preparation of samples for TEM. Samples for TEM were obtained by placing a small amount of each sample on a copper grid. The excess of sample was removed with filter paper and allowed to set for 5 min. A negative stain (1% uranyl acetate) was then added and the samples were left to rest for 30 min before taking the images.

S6.2 Preparation of samples for SEM. Samples for SEM were obtained by freeze drying the gels on copper shim pieces. The freeze-dried samples were then mounted on stubs and the images recorded.

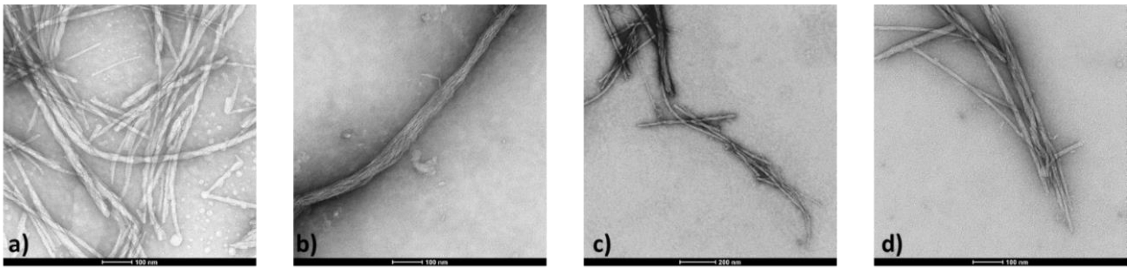

Figure S8. TEM images of (a) DBS-CONHNH$_2$ gel, (b) alginate gel and (c and d) DBS-CONHNH$_2$/alginate two-component gel (interpenetrating networks). Scale bars: 100 nm (a,b,d) and 200 nm (c).

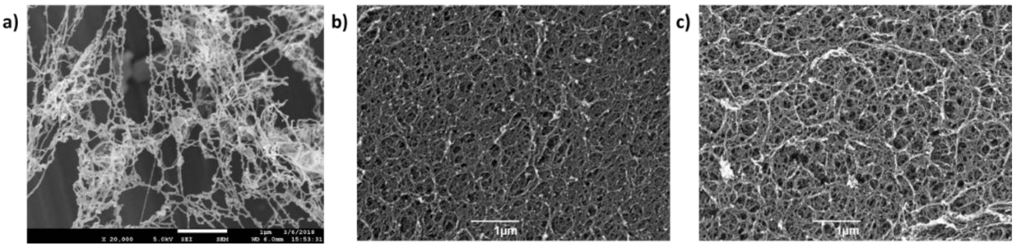

Figure S9. SEM images of (a) DBS-CONHNH$_2$ gel, (b) alginate gel and (c) DBS-CONHNH$_2$/alginate two-component gel (interpenetrating networks). Scale bars: 1 μm.

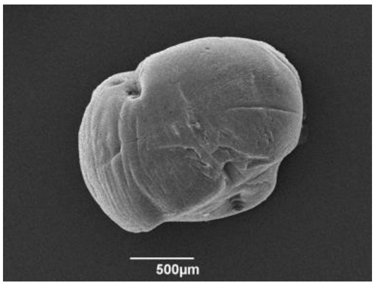

Figure S10. SEM image of DBS-CONHNH$_2$/alginate two-component gel bead. Scale bar: 500 μm.

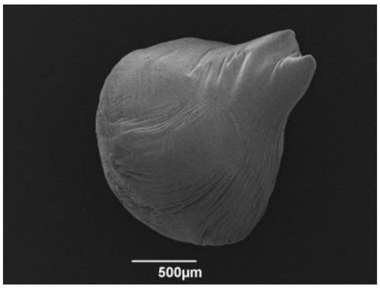

Figure S11. SEM image of alginate gel bead. Scale bar: 500 μm.

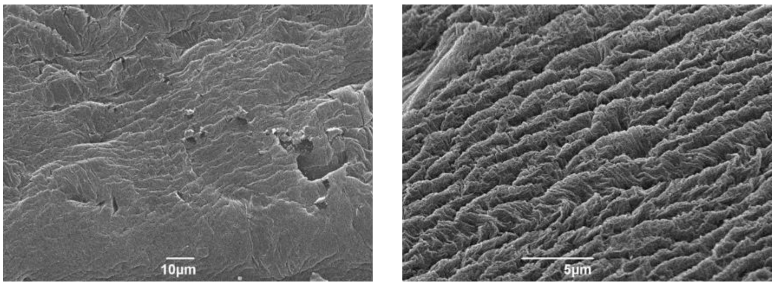

Figure S12. SEM images of DBS-CONHNH$_2$/alginate two-component gel bead surface. Scale bars: 10 μm (left) and 5 μm (right).

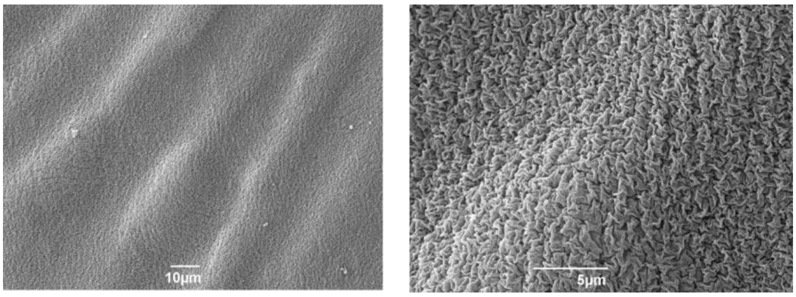

Figure S13. SEM images of alginate gel bead surface. Scale bars: 10 μm (left) and 5 μm (right).

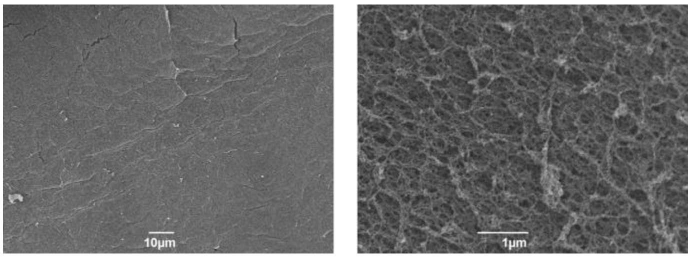

Figure S14. SEM images of alginate gel bead cross-section. Scale bars: 10 μm (left) and 1 μm (right).

S7 Thermal stability studies

All the gels for T_{gel} determination were prepared as described in Section S2 in 2 mL vials (diameter: 1 cm, height: 4 cm). All the gels were placed in a high precision thermoregulated oil bath with an initial temperature of 25°C. The temperature was increased by 1°C/ min until 100°C. Every minute the gels were checked by tube inversion method and T_{gel} was considered as the temperature at which the gel began to run down the sides of the vial. These experiments were performed in triplicate to ensure reproducibility and the average is reported. Errors are estimated at ±2°C.

Table S1. T_{gel} values of gels formed by individual gelators and the DBS-CONHNH$_2$/alginate multicomponent gel (interpenetrating networks).

GEL (1 mL total volume)	LOADING OF DBS-CONHNH$_2$ (wt/vol)	LOADING OF ALGINATE (wt/vol)	T_{gel}
DBS-CONHNH$_2$	0.4%	-	86 °C
Alginate	-	0.4%	>100 °C
Alginate	-	0.6%	>100 °C
Alginate	-	0.8%	>100 °C
DBS-CONHNH$_2$/alginate two-component gel	0.3%	0.1%	>100 °C
DBS-CONHNH$_2$/alginate two-component gel	0.3%	0.3%	>100 °C
DBS-CONHNH$_2$/alginate two-component gel	0.3%	0.5%	>100 °C
DBS-CONHNH$_2$/alginate two-component gel	0.3%	1.0%	>100 °C

S8 Rheology

Gel samples for rheology were prepared as described in Section S2 using bottomless vials as templates to obtain the intended gel dimensions. The measurements were carried out at 25°C using a 20 mm parallel plate and a gap of 2 mm. To avoid solvent evaporation and keep the sample hydrated, a solvent trap was used, and the internal atmosphere was kept saturated. Amplitude sweep experiments were performed in the range of 0.05-100% strain at a 1 Hz frequency to identify the linear viscoelastic region. Frequency sweep experiments were performed between 0.1 and 100 Hz using a shear strain of 0.25%. The measurements were repeated three times to ensure reproducibility and the average data are shown.

Table S2. Rheological data as determined using oscillatory rheometry with a parallel plate geometry, for DBS-CONHNH$_2$ gels, calcium alginate gels, and interpenetrated network gels formed by the combination of the two. Loadings are given in wt/vol, and the G'/G" crossover points refer to the % shear strain at which G"=G'.

Gel	Loading of LMWG	Loading of Alginate	Total Loading	G' (Pa)	G'/G" Crossover
DBS-CONHNH$_2$	0.4%	-	0.4%	800	25.1%
Alginate	-	0.4%	0.4%	490	6.5%
Alginate	-	0.6%	0.6%	1420	19.9%
Alginate	-	0.8%	0.8%	2500	2.3%
Alginate	-	1.3%	1.3%	17100	8.5%
Two-component	0.3%	0.1%	0.4%	1730	50.6%
Two-component	0.3%	0.3%	0.6%	6030	41.2%
Two-component	0.3%	0.5%	0.8%	8030	18.4%
Two-component	0.3%	1.0%	1.3%	17500	2.8%

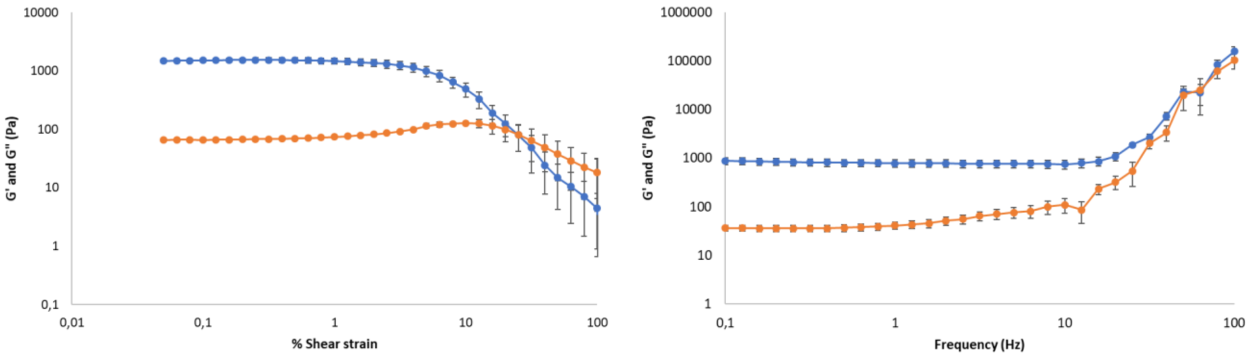

Figure S15. Elastic (G', blue circles) and viscous (G", orange circles) moduli of DBS-CONHNH$_2$ hydrogel (0.4% wt/vol) with increasing shear strain (left) and frequency (right).

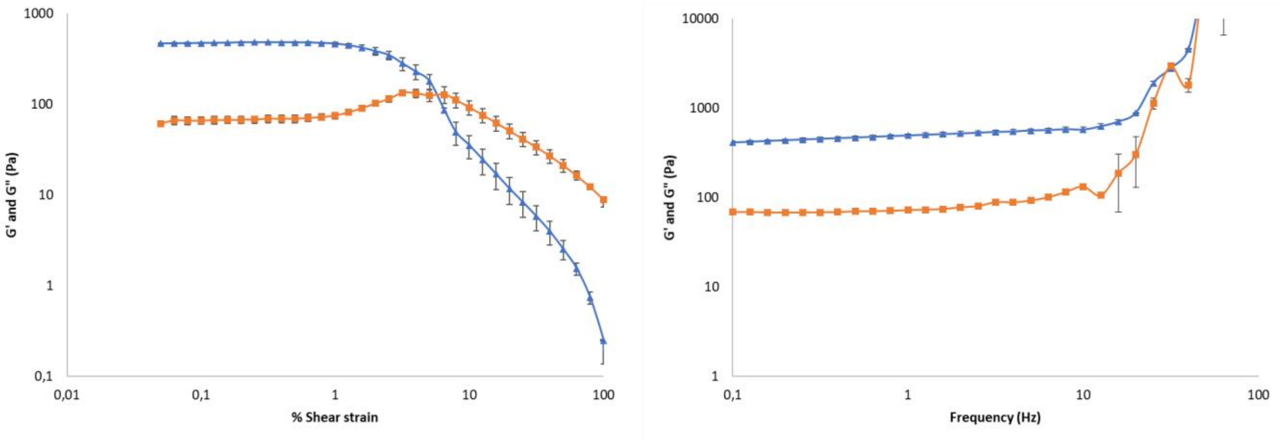

Figure S16. Elastic (G’, blue circles) and viscous (G”, orange circles) moduli of alginate hydrogel (0.4% wt/vol) with increasing shear strain (left) and frequency (right).

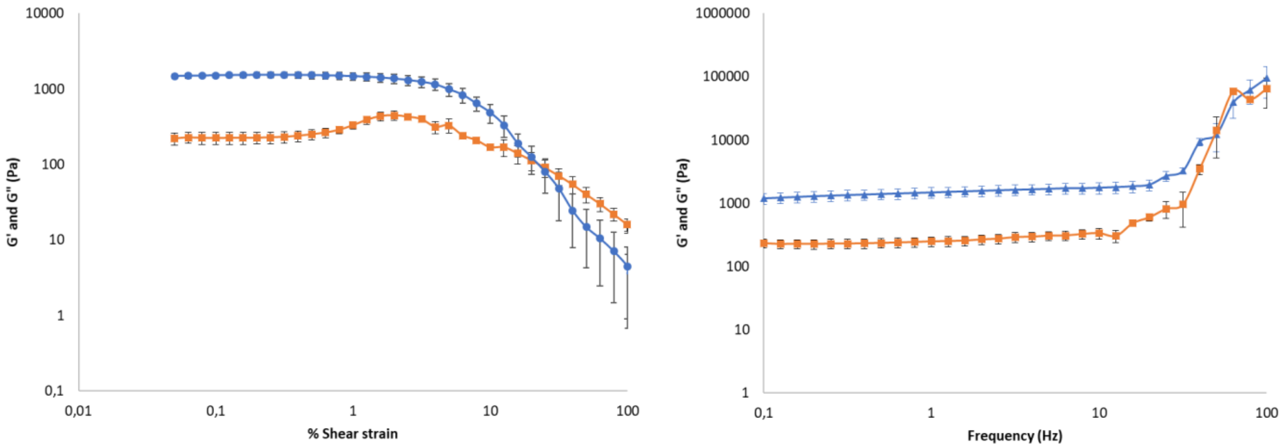

Figure S17. Elastic (G’, blue circles) and viscous (G”, orange circles) moduli of alginate hydrogel (0.6% wt/vol) with increasing shear strain (left) and frequency (right).

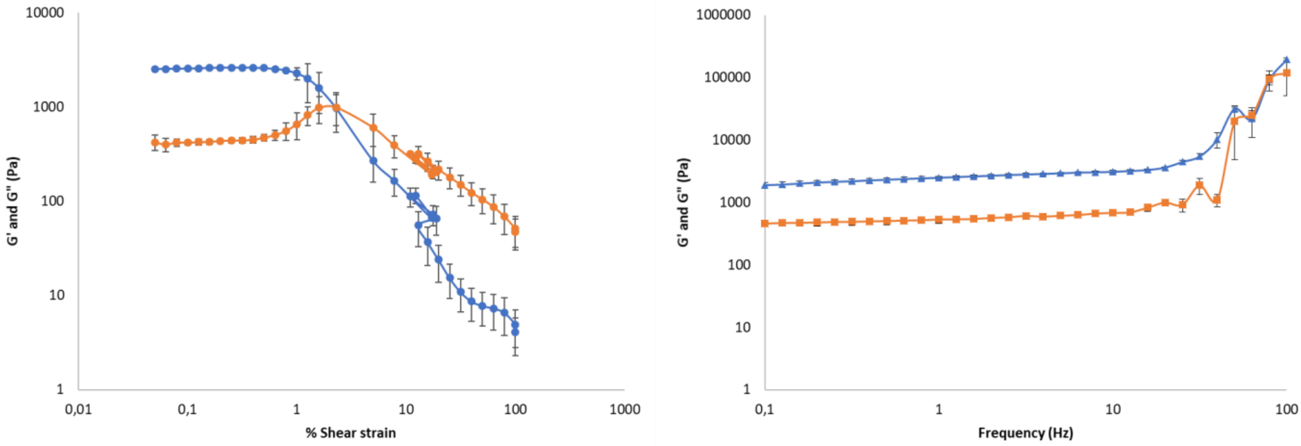

Figure S18. Elastic (G’, blue circles) and viscous (G”, orange circles) moduli of alginate hydrogel (0.8% wt/vol) with increasing shear strain (left) and frequency (right).

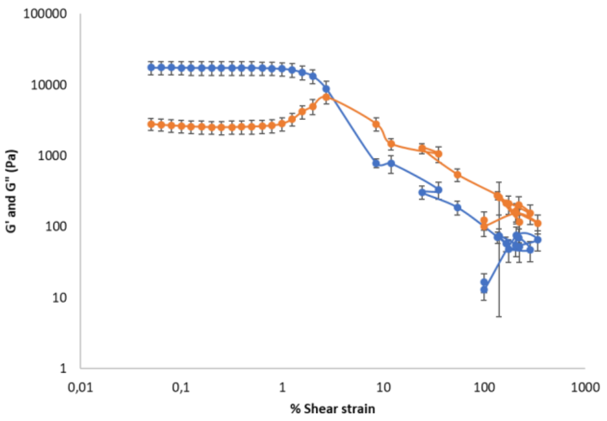

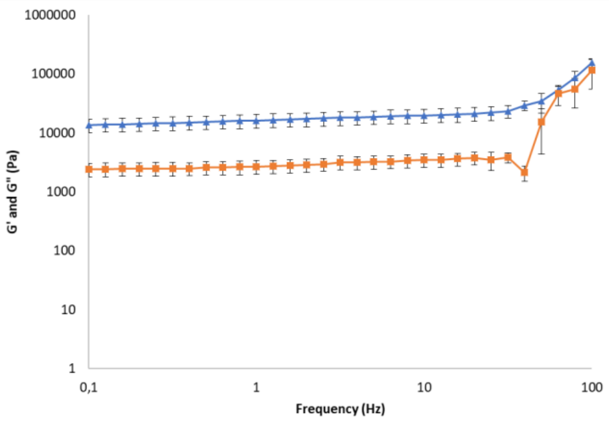

Figure S19. Elastic (G’, blue circles) and viscous (G”, orange circles) moduli of alginate hydrogel (1.3 % wt/vol) with increasing shear strain (left) and frequency (right).

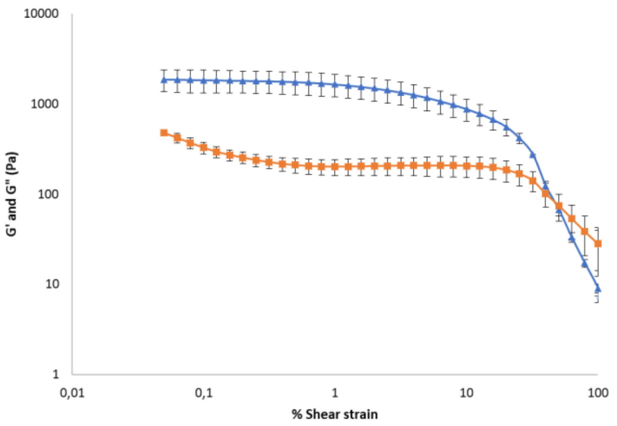

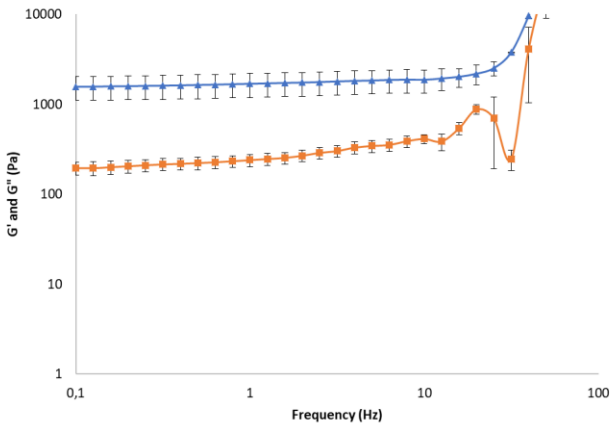

Figure S20. Elastic (G’, blue circles) and viscous (G”, orange circles) moduli of DBS-CONHNH$_2$/alginate two-component hydrogel (0.3% wt/vol DBS-CONHNH$_2$ and 0.1% wt/vol alginate) with increasing shear strain (left) and frequency (right).

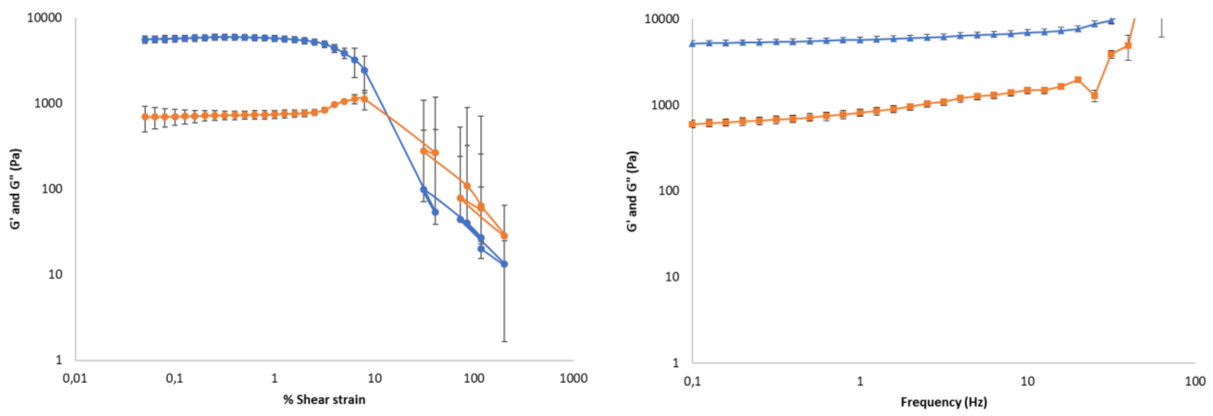

Figure S21. Elastic (G’, blue circles) and viscous (G”, orange circles) moduli of DBS-CONHNH$_2$/alginate multicomponent hydrogel (0.3% wt/vol of both gelators) with increasing shear strain (left) and frequency (right).

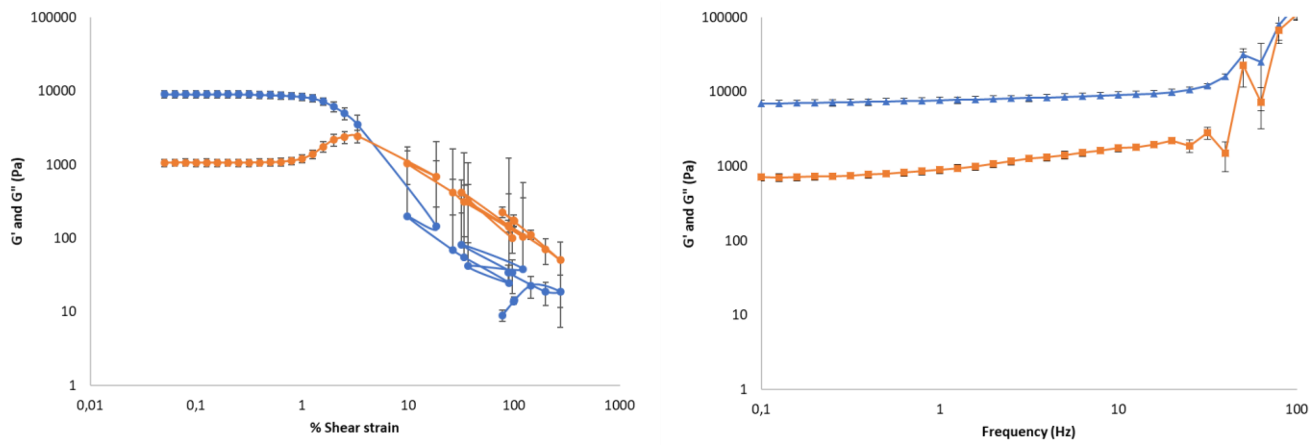

Figure S22. Elastic (G’, blue circles) and viscous (G”, orange circles) moduli of DBS-CONHNH$_2$/alginate multicomponent hydrogel (0.3% wt/vol DBS-CONHNH$_2$ and 0.5% wt/vol alginate) with increasing shear strain (left) and frequency (right).

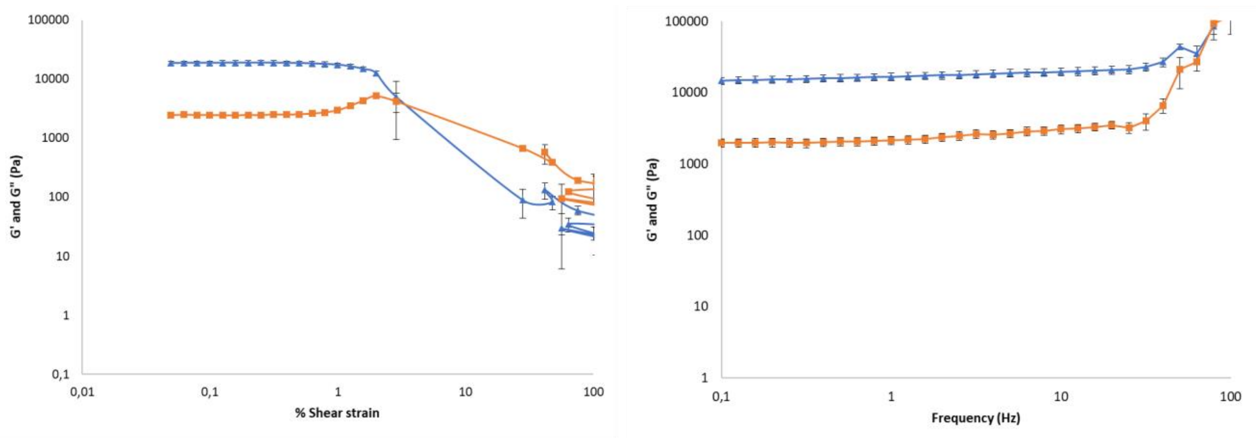

Figure S23. Elastic (G', blue circles) and viscous (G", orange circles) moduli of DBS-CONHNH$_2$/alginate multicomponent hydrogel (0.3% wt/vol DBS-CONHNH$_2$ and 1.0% wt/vol alginate) with increasing shear strain (left) and frequency (right).

S9 Uptake of Pd(II)

S9.1 Uptake Studies by UV-Vis Spectroscopy

Uptake of Pd(II) onto DBS-CONHNH$_2$/alginate multicomponent gels was monitored by UV-VIS spectroscopy and the residual concentration was calculated from the calibration curve plotted for UV-VIS absorption of $PdCl_2$ at λ_{max}= 425 nm.

Table S3. Absorption of standard solution of $PdCl_2$

Sample	Conc. Of $PdCl_2$ [mM]	Absorbance (λ = 425 nm)
1	0.7083	0.20
2	0.3541	0.13
3	0.2361	0.11
4	0.1180	0.083
5	0.0708	0.069
6	0.0472	0.067
7	0.0236	0.059

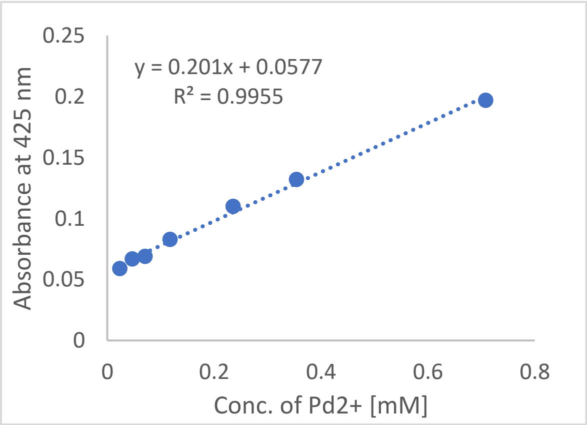

Figure S24. Calibration curve for Pd^{2+} ions.

For the UV studies of Pd uptake, different gel samples were prepared as follows:

Alginate Gel. Prepared according to procedure S2.2 using 0.5 mL of alginate (1 % wt/vol) and 1 mL of $CaCl_2$ (5% wt/vol). The excess of $CaCl_2$ solution was then removed and the gels were washed with water multiple times.

Extended Interpenetrating Network Two-Component Gel. DBS-$CONHNH_2$ gel was prepared using 3.00 mg of DBS-$CONHNH_2$ suspended in water (0.5 mL). Alginate solution (1% wt/vol, 0.5 mL) was placed on the top of the gel and left to diffuse for 3 days. After three days, the upper liquid was carefully removed with Pasteur pipette, and $CaCl_2$ solution (5% wt/vol, 1 mL) was placed on top of the gel and left to diffuse overnight. The excess of $CaCl_2$ solution was then removed and the gels were washed with water multiple times.

Two-Component Gel Beads. Prepared according to procedure S2.4 using 3.00 mg of DBS-$CONHNH_2$ suspended in water (0.5 mL), 0.5 mL of alginate (1% wt/vol) and 1 mL of $CaCl_2$ (5% wt/vol). The excess of $CaCl_2$ solution was then removed and the gels were washed with water multiple times.

Each of these gels was immersed in 3 mL of $PdCl_2$ solution (approx. 5 mM, the precise concentration was determined by UV before Pd uptake) and left to interact. At specified times, the solutions were analysed by UV and then transferred back to the gels.

Table S4. Amount of Pd taken up by the alginate gel beads.

t (h)	Absorbance (λ = 425nm)	Amount of Pd in gel [µmol]
**0**	1.164	0.00
**0.5**	0.828	4.96
**1**	0.713	6.67
**2**	0.679	7.18
**4**	0.665	7.39
**24**	0.638	7.79
**48**	0.638	7.79
**72**	0.638	7.79
**195**	0.606	8.27

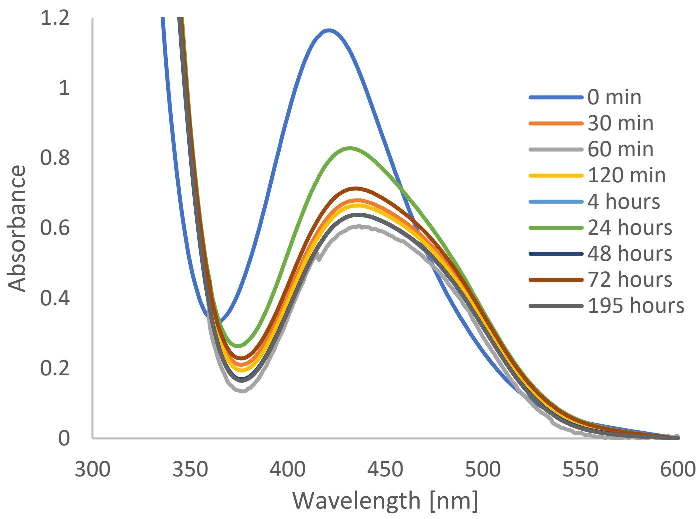

Figure S25. UV-Vis spectra of $PdCl_2$ solution after interaction with alginate gel beads at room temperature

Table S5. Amount of Pd within the two-component extended interpenetrating network gel

t (h)	Absorbance (λ = 425 nm)	Amount of Pd in gel [µmol]
**0**	1.122	0.00
**0.5**	0.927	3.48
**1**	0.834	4.87
**2**	0.719	6.58
**4**	0.618	8.09
**6**	0.580	8.66
**23**	0.510	9.70
**48**	0.487	10.04
**72**	0.478	10.18
**144**	0.457	10.49

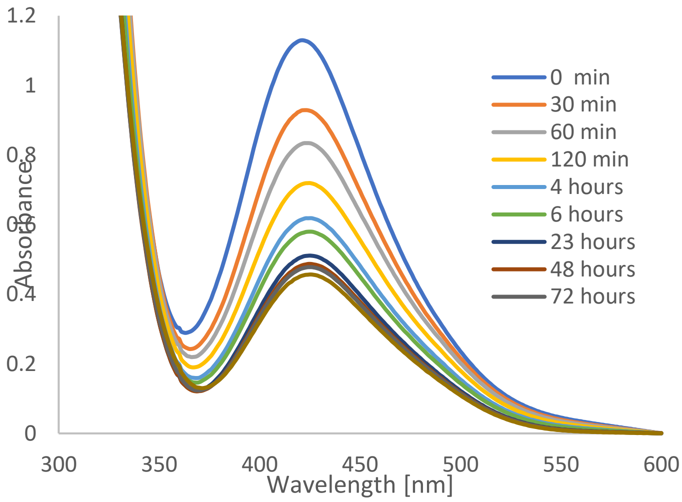

Figure S26. UV-Vis spectra of $PdCl_2$ solution after interaction with two-component interpenetrating network gel at room temperature

Table S6. Amount of Pd taken up by the two-component gel beads.

t (h)	Absorbance (λ = 425 nm)	Amount of Pd in gel [μmol]
**0**	1.160	0.00
**0.5**	0.785	5.60
**1**	0.646	7.67
**2**	0.600	8.36
**4**	0.559	8.97
**6**	0.542	9.22
**24**	0.483	10.10
**48**	0.465	10.37
**72**	0.457	10.49
**197**	0.437	10.79

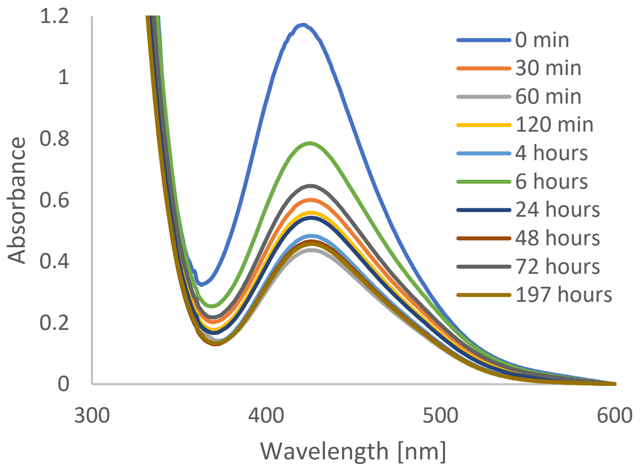

Figure S27. UV-Vis spectra of $PdCl_2$ solution after interaction with two-component gel beads at room temperature

S10.2 TEM and SEM images of two-component gel spheres loaded with PdNPs

a) b)

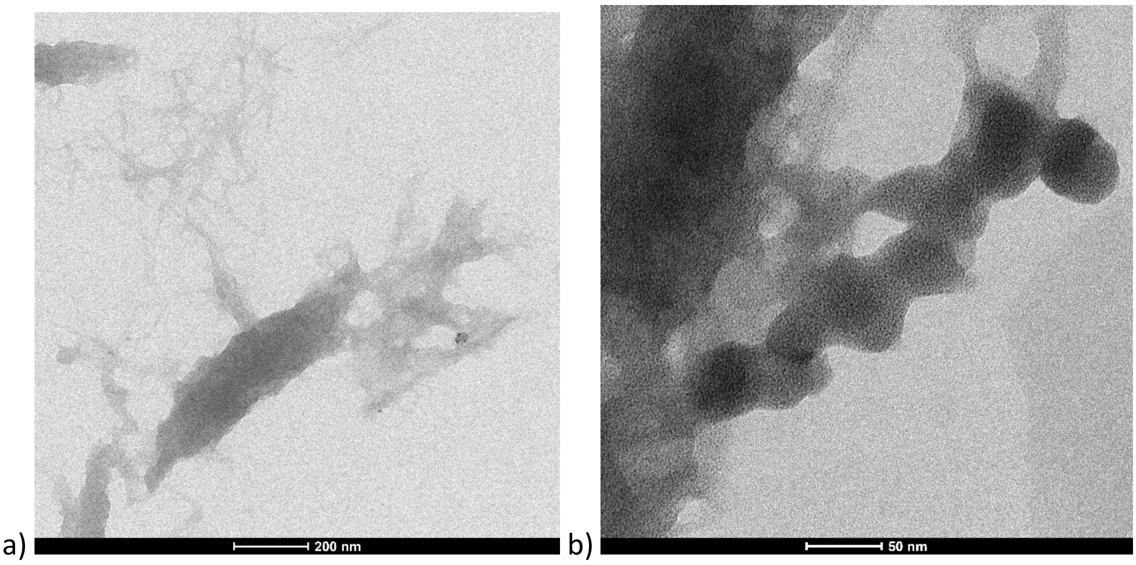

Figure S28. TEM images of two-component gel spheres loaded with PdNPs, scale bars: a) 200 nm; b) 50 nm.

a) 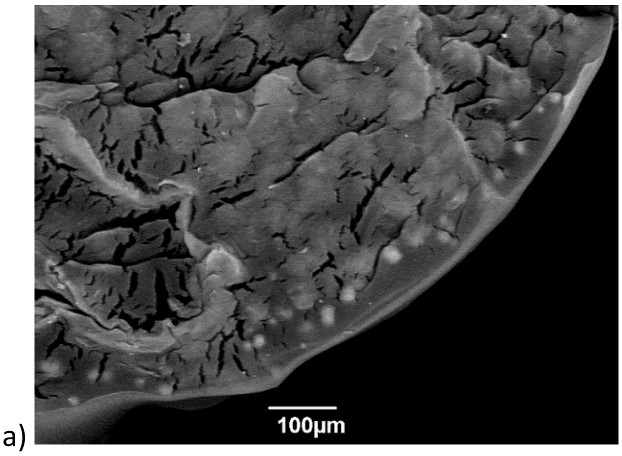

b) 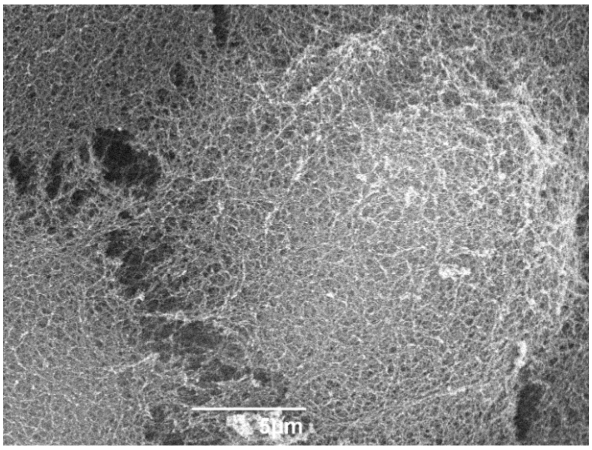

c) 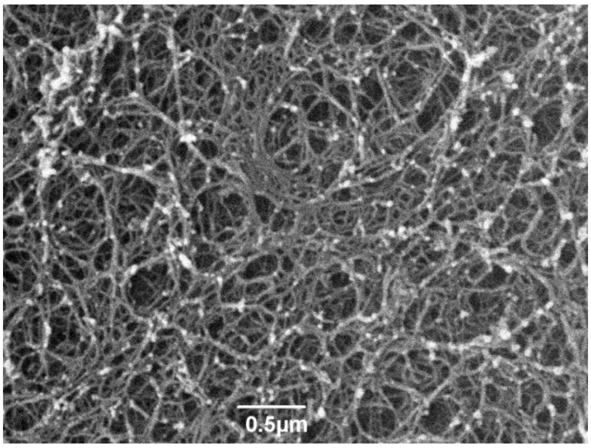

Figure S29. SEM images of two-component gel spheres loaded with PdNPs, scale bars: a) 100 µm; b) 5 µm; c) 0.5 µm.

a) 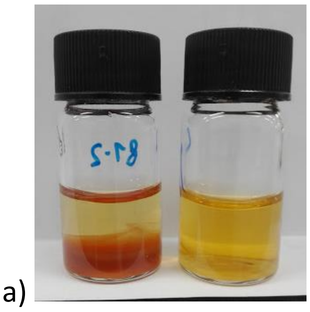
b) 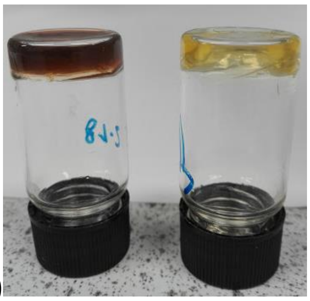

Figure S30. Comparison of colour change during Pd uptake between (left) two-component gel and (right) alginate gel after: a) 4 hours; b) 169 hours.

S10 Suzuki-Miyaura cross-coupling reaction

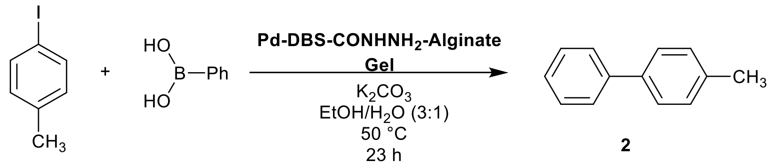

Scheme S1. Suzuki-Miyaura reaction investigated in this paper.

S10.1 Reaction catalysed by extended interpenetrated two-component gel. 4-Iodotoluene (1 mmol), phenylboronic acid (1.2 mmol) and K_2CO_3 (2 mmol) was dissolved in 4 mL of $EtOH/H_2O$ mixture (3:1) in a reaction vial. To this mixture, the DBS-$CONHNH_2$/alginate gel (made from 3.00 mg of DBS-$CONHNH_2$ in 0.5 mL H_2O, 0.5 mL 1% alginate, 1 mL 5% $CaCl_2$) containing approx. 12 µmol of Pd (1.2%) was carefully added. The reaction vial was heated to 50°C and left to react with stirring for 23 hours. Progress of the reaction was monitored by TLC. The gel was destroyed during the reaction. After the reaction was complete, product was extracted with diethyl ether and washed with 1 M NaOH and water. The organic phase was dried over $MgSO_4$ and solvent was evaporated under reduced pressure to give the desired product **2** in 98% yield as a white powder. The recorded spectroscopic data correlate with those reported in literature.[3]

S10.2 Reaction catalysed by a single two-component gel sphere. 4-Iodotoluene (0.8 mmol), phenylboronic acid (0.96 mmol) and K_2CO_3 (1.6 mmol) was dissolved in 4 mL of $EtOH/H_2O$ mixture (3:1) in a reaction vial. To this mixture, the DBS-$CONHNH_2$/alginate gel sphere (prepared according to S2.4) containing approx. 0.4 µmol of Pd (0.05%) was carefully added. The reaction vial was heated to 50°C and left to react without stirring for 22 hours. Progress of the reaction was monitored by TLC. The gel sphere was partly destroyed during the reaction. After the reaction was complete, product was extracted with diethyl ether and washed with 1 M NaOH and water. The organic phase was dried over $MgSO_4$ and solvent was evaporated under reduced pressure to give the desired product **2** in 99% yield as a white powder. The recorded spectroscopic data correlate with those reported in literature.[3]

S10.3 Recycling studies. 4-Iodotoluene (0.8 mmol), phenylboronic acid (0.96 mmol) and K_2CO_3 (1.6 mmol) was dissolved in 4 mL of $EtOH/H_2O$ mixture (3:1) in a reaction vial. To this mixture, the DBS-$CONHNH_2$/alginate gel sphere (prepared according to S2.4) containing approx. 0.4 µmol of Pd

(0.05%) was carefully added. The reaction vial was heated to 50°C and left to react without stirring for 24 hours. Progress of the reaction was monitored by TLC. The gel sphere got partly destroyed during the reaction. After the reaction was complete, the solvent was carefully extracted with Pasteur pipette leaving the gel bead in the reaction vial. The reaction vial was further washed with diethyl ether and water. The reaction vial containing the gel bead was charged with new starting materials and the reaction was performed again. The reaction mixtures were treated as in previous experiments (S11.2). The conversions were determined by ^{1}H NMR by comparing integral values of CH_3 groups of starting material and product.

S10.4 General procedure for Suzuki-Miyaura cross-coupling reaction using a single two-component gel sphere. Aryl iodide (0.8 mmol), phenylboronic acid (0.96 mmol) and K_2CO_3 (1.6 mmol) was dissolved in 4 mL of EtOH/H_2O mixture (3:1) in a reaction vial. To this mixture, the DBS-CONHNH_2/alginate gel sphere (prepared according to S2.4) containing approx. 0.4 µmol of Pd (0.05%) was carefully added. The reaction vial was heated to 50°C and left to react without stirring for 24 hours. Progress of the reaction was monitored by TLC. After the reaction was complete, product was extracted with diethyl ether and washed with 1 M NaOH and water. The organic phase was dried over $MgSO_4$ and solvent was evaporated under reduced pressure to give the desired product.

S10.5 Characterization data of products

4-Methylbiphenyl (2a): white solid. ^{1}H NMR ($CDCl_3$, 400 MHz, 293 K): δ = 7.66-7.58 (m, 2H), 7.56-7.51 (m, 2H), 7.49-7.42 (m, 2H), 7.39-7.32 (m, 1H), 7.31-7.26 (m, 2H), 2.43 (s, 3H) ppm. The recorded spectroscopic data correlate with those reported in literature.[3]

4-Methoxybiphenyl (2b)

White solid, 0.1462 g (99%, full conversion). ^{1}H NMR ($CDCl_3$, 400 MHz, 293 K): δ 7.59 – 7.50 (m, 4H), 7.42 (t, J = 7.7 Hz, 2H), 7.35 – 7.27 (m, 1H), 7.03 – 6.93 (m, 2H), 3.85 (s, 3H) ppm. The recorded spectroscopic data correlate with those reported in literature.[3]

3,4-Dimethoxybiphenyl (2c)

White solid, 0.1653 g (91%, conversion 94%). ^{1}H NMR ($CDCl_3$, 400 MHz, 293 K): δ = 7.58 – 7.53 (m, 2H), 7.45 – 7.39 (m, 2H), 7.34 – 7.28 (m, 1H), 7.17 – 7.10 (m, 2H), 6.94 (d, 1H, *J* = 8.3 Hz), 3.95 (s, 3H), 3.92 (s, 3H) ppm. The recorded spectroscopic data correlate with those reported in literature.[3]

4-Fluorobiphenyl (2d)

White solid, 0.1252 g (91%, full conversion). ^{1}H NMR ($CDCl_3$, 400 MHz, 293 K): δ = 7.57 – 7.50 (m, 4H), 7.46 – 7.39 (m, 2H), 7.37 – 7.30 (m, 1H), 7.15 – 7.07 (m, 2H) ppm. The recorded spectroscopic data correlate with those reported in literature.[3]

4-Nitrobiphenyl (2e)

White solid, 0.1578 g (99%, full conversion). ^{1}H NMR ($CDCl_3$, 400 MHz, 293 K): δ = 8.33 – 8.26 (m, 2H), 7.76 – 7.70 (m, 2H), 7.64 – 7.59 (m, 2H), 7.53 – 7.40 (m, 3H) ppm. The recorded spectroscopic data correlate with those reported in literature.[3]

2-Phenylthiophene (2f)

White solid, 0.1106 g (86%, full conversion). ^{1}H NMR ($CDCl_3$, 400 MHz, 293 K): δ = 7.62 (d, *J* = 8.1 Hz, 2H), 7.38 (t, *J* = 7.6 Hz, 2H), 7.34 – 7.26 (m, 4H), 7.12 – 7.06 (m, 1H) ppm. The recorded spectroscopic data correlate with those reported in literature.[4]

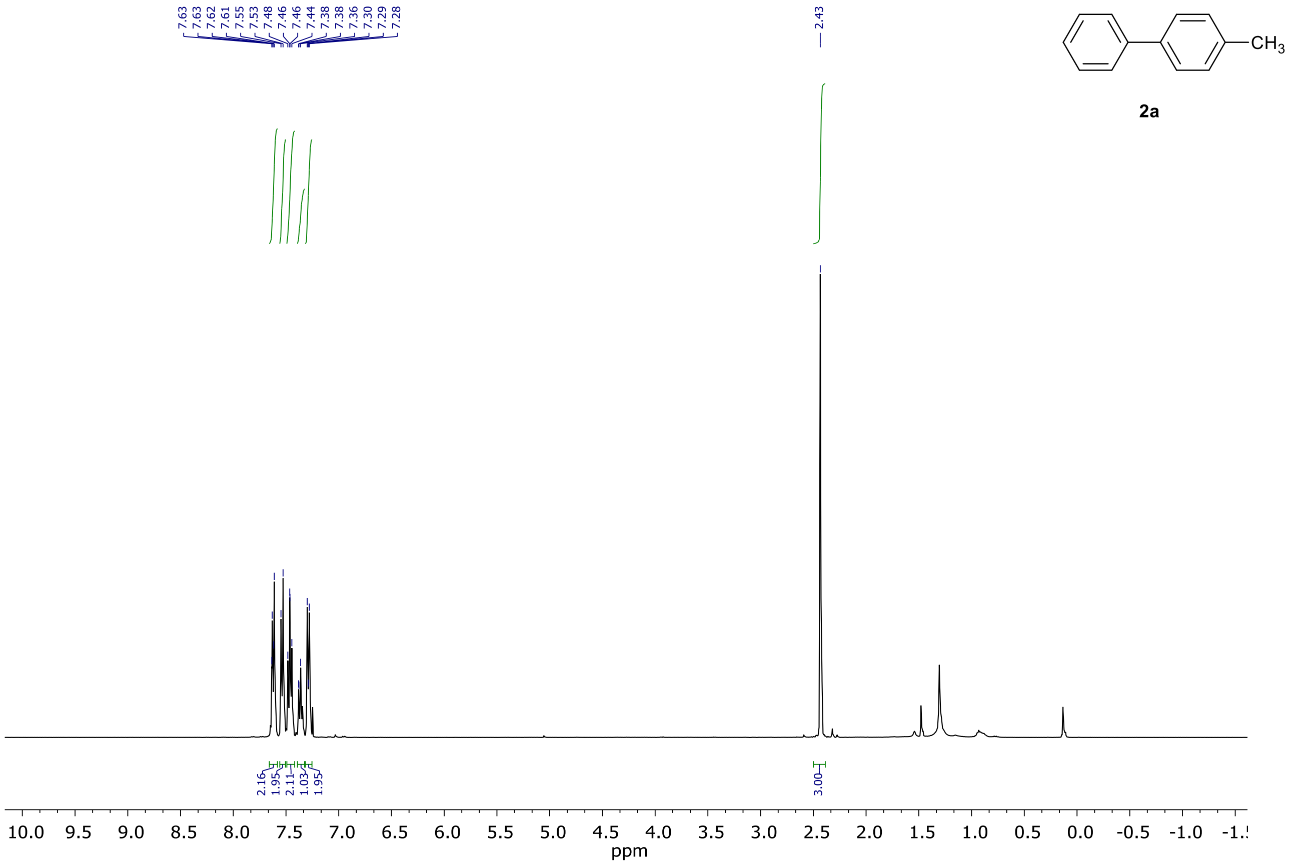

Figure S31. ^{1}H NMR of compound **2a** ($CDCl_3$, 400 MHz).

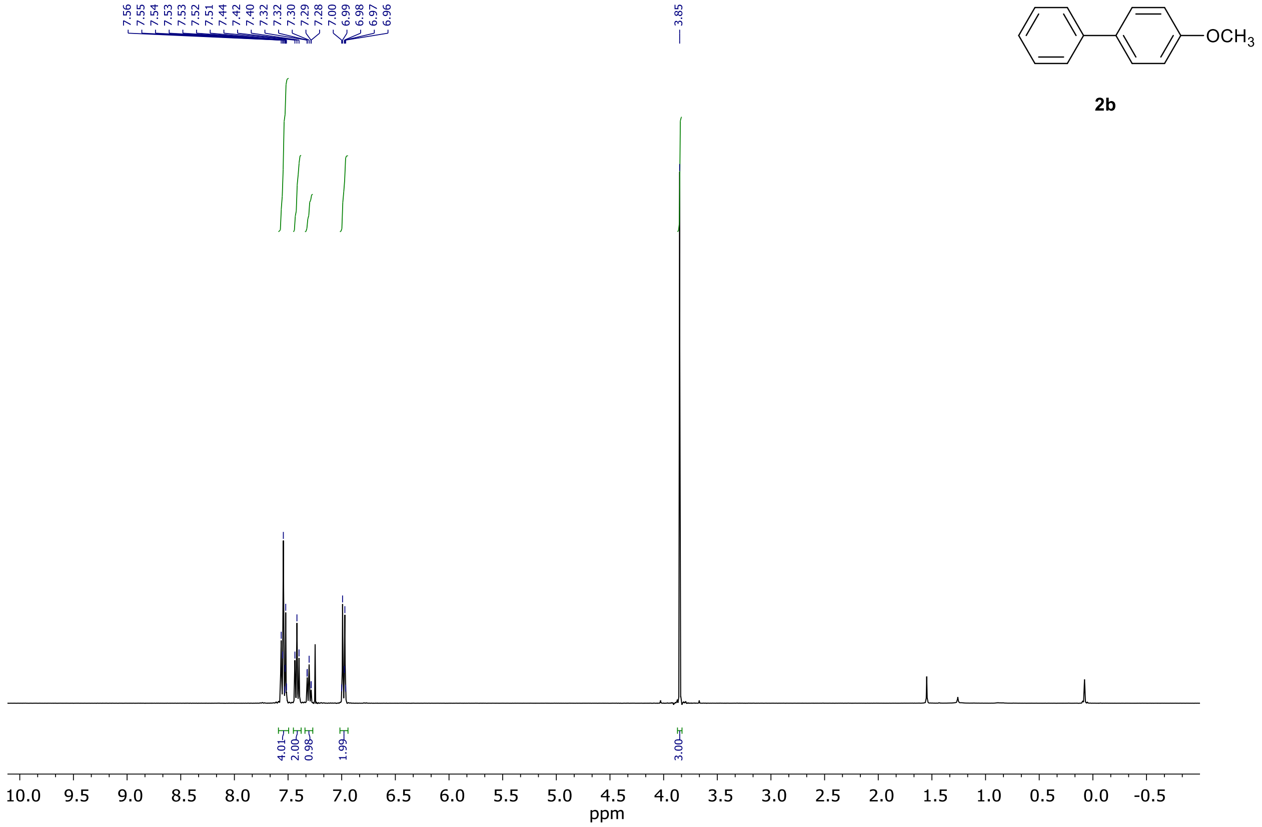

Figure S32. ^{1}H NMR of compound **2b** ($CDCl_3$, 400 MHz).

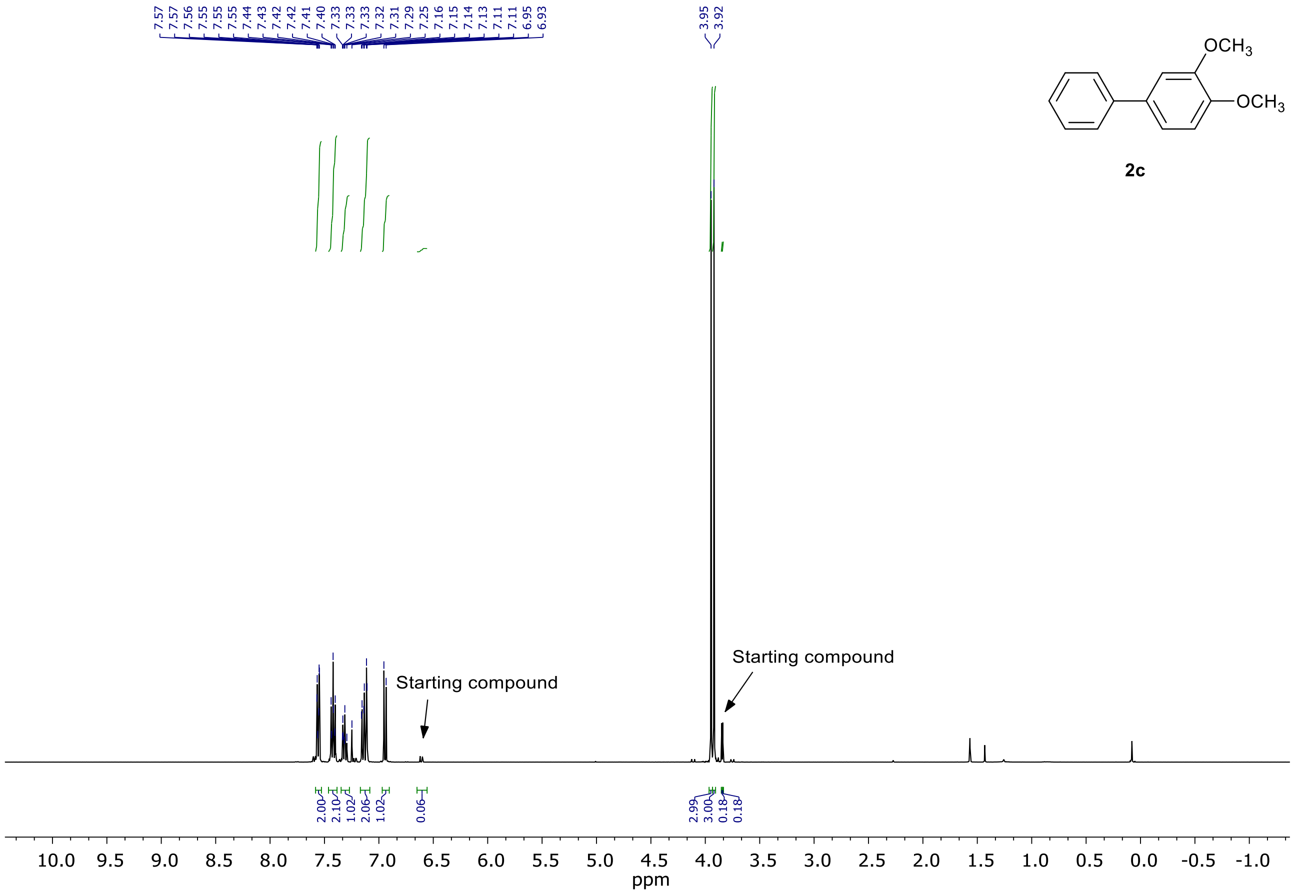

Figure S33. ^{1}H NMR of compound **2c** ($CDCl_3$, 400 MHz).

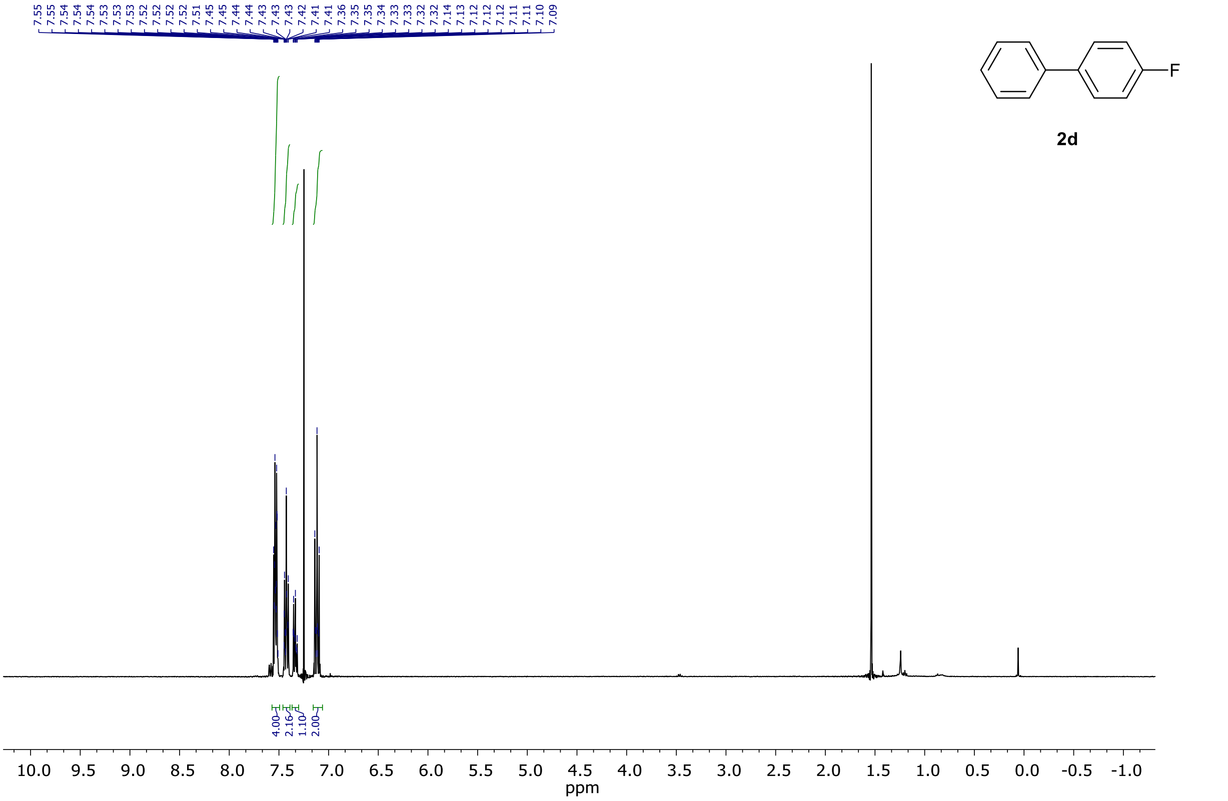

Figure S34. ^{1}H NMR of compound **2d** ($CDCl_3$, 400 MHz).

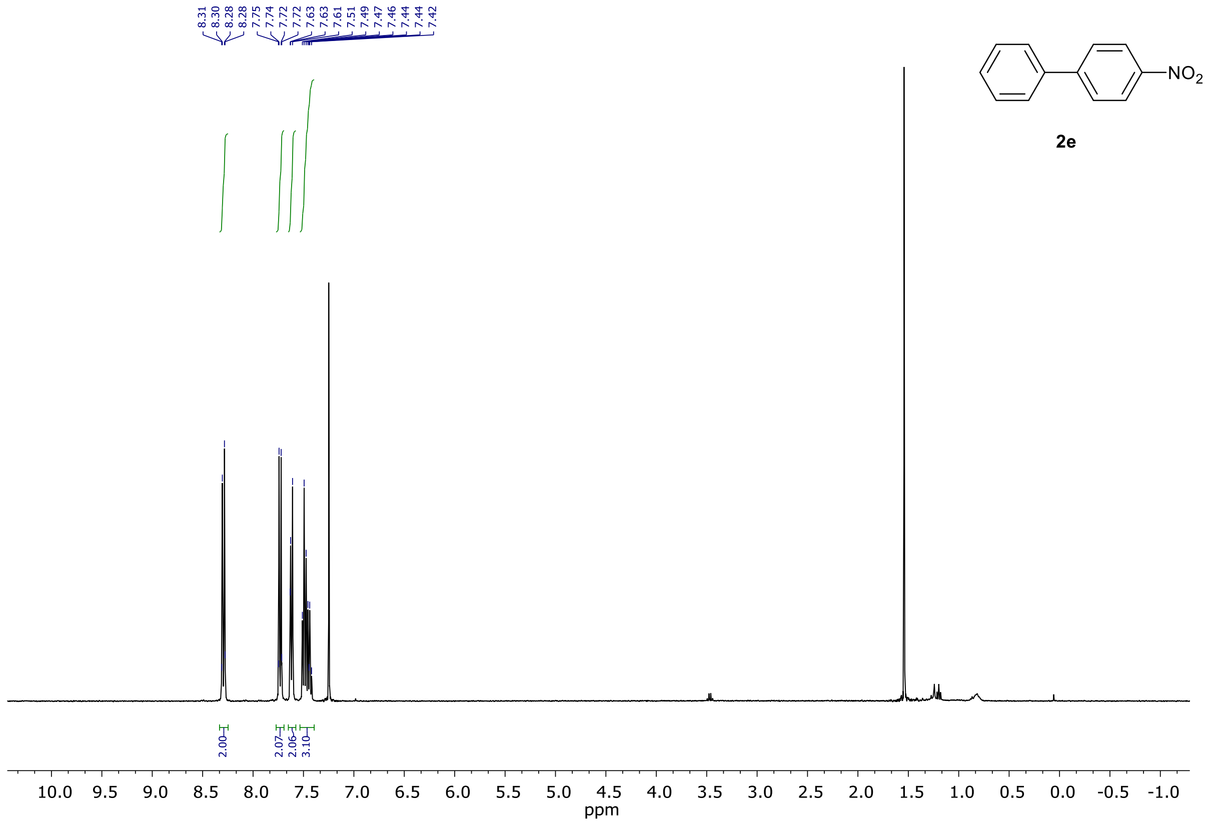

Figure S35. ^{1}H NMR of compound **2e** ($CDCl_3$, 400 MHz).

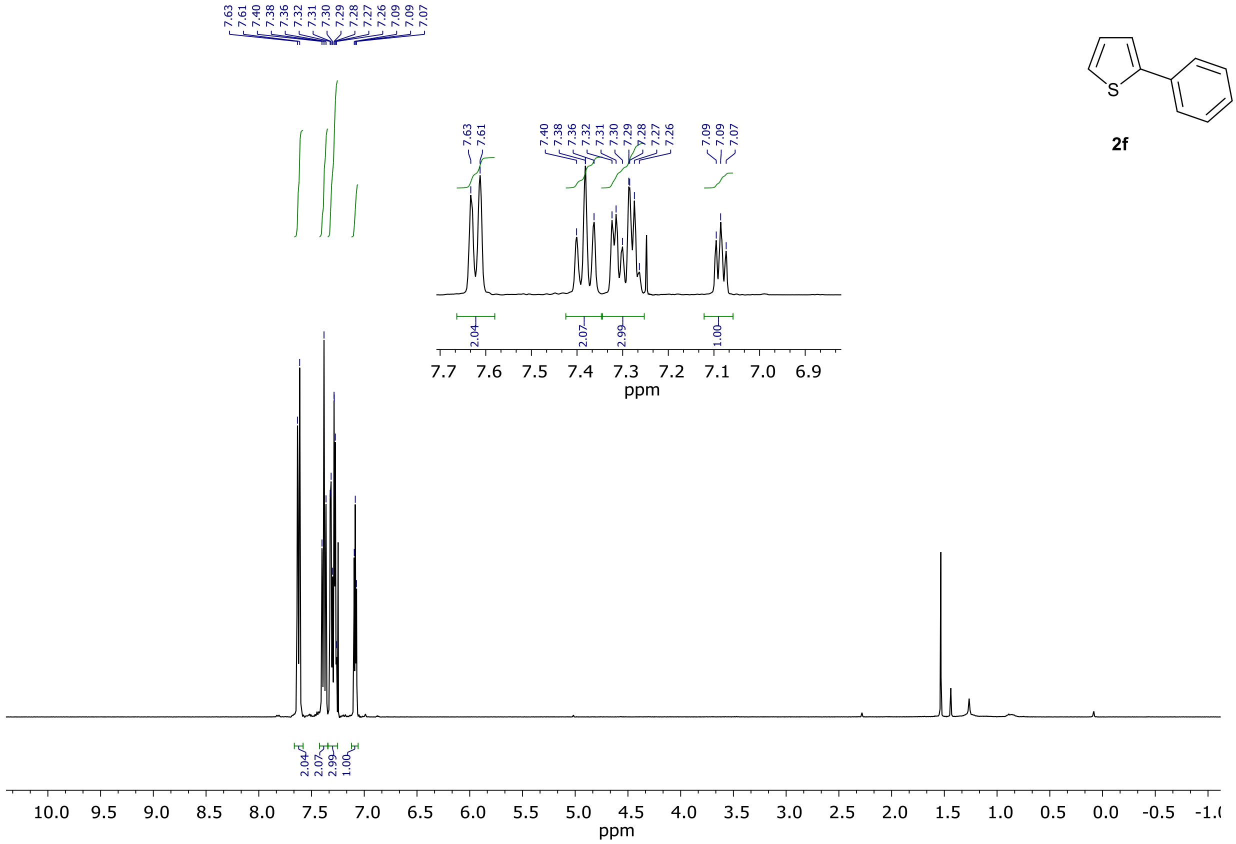

Figure S36. ^{1}H NMR of compound **2f** ($CDCl_3$, 400 MHz).

S11 Pd Leaching Experiment

4-Iodotoluene (0.8 mmol), phenylboronic acid (0.96 mmol) and K_2CO_3 (1.6 mmol) were dissolved in a 3:1 mixture of ethanol and water (4 mL). To this mixture, a DBS-CONHNH$_2$ /alginate gel sphere (prepared according to S2.4) containing approx. 0.4 μmol of Pd (0.05%) was carefully added. The reaction vial was heated to 50°C and left to react without stirring for 24 hours. After 24 hours, the gel sphere was removed, and the hot reaction mixture, containing product 2a, was filtered through a nylon syringe filter (0.22 μm) into a new vial. To this solution, 4-iodoanisole (0.8 mmol), phenylboronic acid (0.96 mmol) and K_2CO_3 (1.6 mmol) was added and another Suzuki cross-coupling reaction was performed in the absence of the gel bead with the same described conditions to give product **2b**. After 24 hours, the crude reaction mixture was analysed by ^{1}H NMR to determine reaction conversion. Although the reaction was carried out after removal of the gel bead, approximately 96 % conversion was observed for product **2b**, suggesting that some Pd leached out from the gel sphere during the first reaction (product **2a** - full conversion), catalysing the second one.

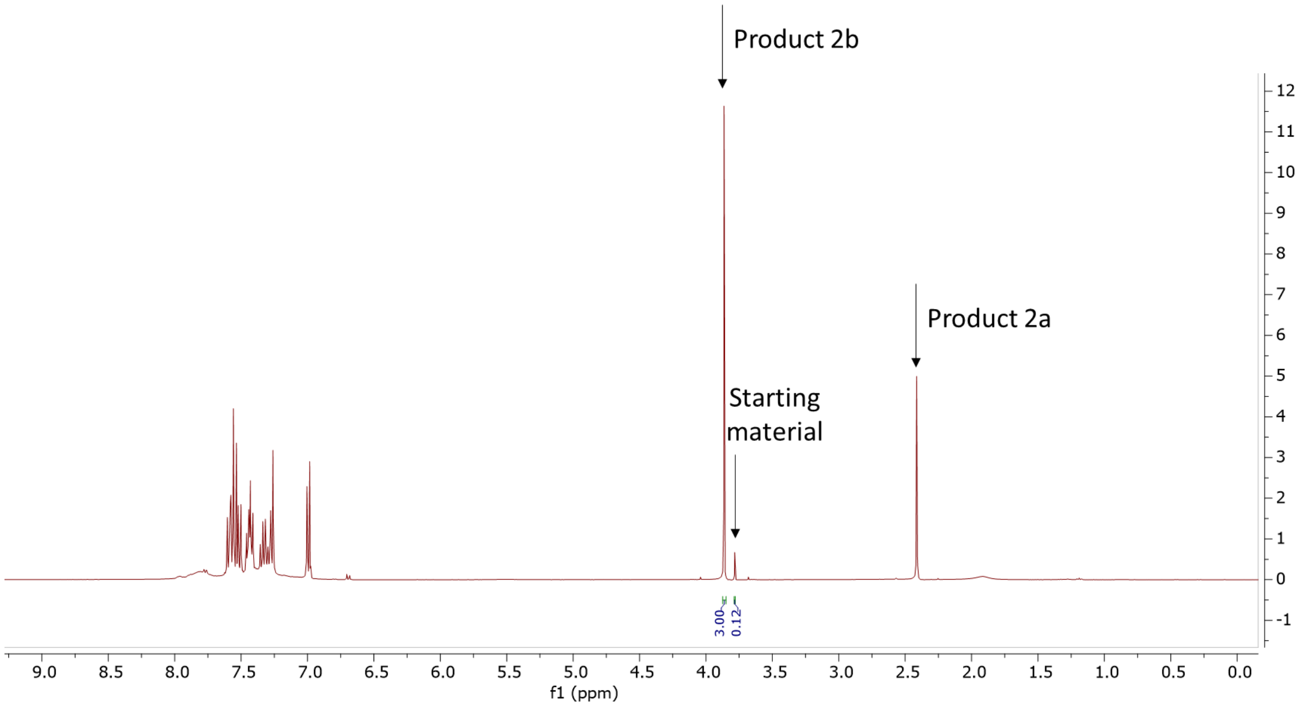

Figure S37. ^{1}H NMR of compound **2b** ($CDCl_3$, 400 MHz) formed in the absence of the gel nanosphere after Pd leaching during the formation of **2a**.

S12 References

[1] B. O. Okesola, D. K. Smith, *Chem. Commun.*, **2013**, *49*, 11164-11166.

[2] D. J. Cornwell, B. O. Okesola, D. K. Smith, *Soft Matter*, **2013**, *9*, 8730-8736.

[3] F. Mäsing, H. Nüsse, J. Klingauf, A. Studer, *Org. Lett.*, **2018**, *20*, 752-755.

[4] L. Zhang, Z. Su, F. Jiang, Y. Zhou, W. Xu, M. Hong, *Tetrahedron*, **2013**, *69*, 9237−9244.
